# A Novel 3-Hydroxysteroid Dehydrogenase That Regulates Reproductive Development and Longevity

**DOI:** 10.1371/journal.pbio.1001305

**Published:** 2012-04-10

**Authors:** Joshua Wollam, Daniel B. Magner, Lilia Magomedova, Elisabeth Rass, Yidong Shen, Veerle Rottiers, Bianca Habermann, Carolyn L. Cummins, Adam Antebi

**Affiliations:** 1 Department of Molecular and Cellular Biology, Huffington Center on Aging, Baylor College of Medicine, Houston, Texas, United States of America; 2 Interdepartmental Program in Cell and Molecular Biology, Baylor College of Medicine, Houston, Texas, United States of America; 3 Max Planck Institute for Biology of Ageing, Cologne, Germany; 4 Department of Pharmaceutical Sciences, University of Toronto, Toronto, Ontario, Canada; 5 Cologne Excellence Cluster on Cellular Stress Responses in Aging Associated Diseases (CECAD), University of Cologne, Cologne, Germany; Massachusetts General Hospital Havard Medical School, United States of America

## Abstract

Endogenous small molecule metabolites that regulate animal longevity are emerging as a novel means to influence health and life span. In *C. elegans*, bile acid-like steroids called the dafachronic acids (DAs) regulate developmental timing and longevity through the conserved nuclear hormone receptor DAF-12, a homolog of mammalian sterol-regulated receptors LXR and FXR. Using metabolic genetics, mass spectrometry, and biochemical approaches, we identify new activities in DA biosynthesis and characterize an evolutionarily conserved short chain dehydrogenase, DHS-16, as a novel 3-hydroxysteroid dehydrogenase. Through regulation of DA production, DHS-16 controls DAF-12 activity governing longevity in response to signals from the gonad. Our elucidation of *C. elegans* bile acid biosynthetic pathways reveals the possibility of novel ligands as well as striking biochemical conservation to other animals, which could illuminate new targets for manipulating longevity in metazoans.

## Abstract 2

A multidisciplinary approach identifies novel biochemical activities involved in the synthesisof *C. elegans* bile acid-like steroids, which act as hormones that regulate sterol metabolism and longevity.

## Author Summary

Although well known for their role in the absorption of dietary fat, bile acids have emerged as important metabolic signaling molecules that regulate cholesterol, fat, and glucose metabolism. Bile acids work through nuclear receptors, a class of transcription factors that bind to fat soluble hormones to directly control target gene expression. In the roundworm *C. elegans*, DAF-12 is a nuclear receptor for bile acids, called the dafachronic acids, which are known to regulate development and longevity, however the synthesis and regulation of these molecules remain unclear. Here we identify novel biochemical activities, including a conserved 3-hydroxysteroid dehydrogenase, involved in the production of the dafachronic acids, illuminating their role in cholesterol and bile acid metabolism, and longevity. The identified activities reveal remarkable evolutionary conservation to those seen in mammalian bile acid synthesis, potentially providing novel ways to manipulate animal lifespan and cholesterol homeostasis.

## Introduction

Small molecule metabolites and peptide hormones are well known to regulate various aspects of animal physiology, metabolism, and homeostasis. More recently they have emerged as important modulators of life span. Notably, a modest reduction in insulin/IGF-1 signaling (IIS) has been shown to increase longevity in several organisms from worms to mice and possibly humans [1]. Several naturally occurring metabolites including spermidine, trehalose, endocannabinoids, and oleic acid are among a handful of small molecules impacting longevity in model systems [2]–[5]. Metabolites of cholesterol, such as steroids, oxysterols, and bile acids, act through cognate nuclear hormone receptor transcription factors (NHRs) to regulate gene expression [6],[7]. Of these sterol metabolites, bile acids are primarily known for their roles in dietary fat absorption, but are increasingly recognized as important signaling molecules, regulating aspects of cholesterol, glucose, and fatty acid metabolism through the control of sterol-sensing NHRs, including FXRα, LXR, and G-protein coupled receptors [8]–[10].

In *C. elegans*, DAF-12, a homolog of FXRα and LXR, is activated by the bile acid-like dafachronic acids (DAs) and governs key events that influence longevity [11],[12]. In particular, DAF-12 regulates the decision to undergo reproductive development or arrest at the dauer diapause, an alternative developmental stage characterized by stress resistance and extended longevity [12]–[14]. DAF-12 also regulates adult longevity in response to signals from the gonad. Loss of the germline through laser microsurgery or genetic manipulation leads to an extended lifespan, dependent upon DAF-12 and its ligands [1],[15],[16]. The discovery of endogenous ligands for DAF-12, which include Δ^4^- and Δ^7^-dafachronic acids and the structurally related 25-*S*-cholestenoic acid, provided the first evidence that bile acid-like molecules modulate animal lifespan [11],[17],[18].

Molecular and genetic studies indicate that an endocrine network governs dauer formation and longevity. These experiments suggest a model whereby environmental signals indicating favorable conditions are integrated *via* neurosensory processing, which stimulate IIS and TGF-β signaling. These pathways converge to activate DAF-12 by promoting DA biosynthesis, thus facilitating growth to reproductive maturity and a normal lifespan. Conversely, in unfavorable conditions, upstream endocrine pathways are downregulated and are thought to decrease DA levels. In the absence of its ligands, DAF-12 associates with the co-repressor DIN-1/SHARP and promotes entry into the long-lived dauer stage [12],[19]–[21]. Thus, DA availability regulates DAF-12 activity, controlling the binary dauer decision and lifespan, but how DA availability is achieved is not well understood. In addition, DAF-12 and DA signaling play a conserved role in regulating nematode dauer formation across wide evolutionary distances, including in parasitic nematodes whose infective stages are analogous to dauer [22],[23]. Treatment with DA promotes exit from diapause in these animals, suggesting that components of DA biosynthesis and DAF-12 signaling are potential anti-helminthic therapeutic targets.

Although DA availability controls DAF-12 activity to influence longevity, the synthesis and regulation of these molecules are poorly understood. The DAs are derived from dietary cholesterol, which is required for nematode viability and fertility. Previous studies identified DAF-36, a Rieske-like oxygenase with cholesterol 7-desaturase activity; DAF-9, a cytochrome P450 with activity similar to mammalian CYP27A1; and HSD-1, a putative 3-β-hydroxysteroid dehydrogenase homolog as acting in the biosynthesis of DAs from dietary cholesterol [11],[18],[24]–[29]. However, the nodes of regulation by upstream signaling pathways, environmental and nutritional signals, as well as the functional relationship of identified gene products, intermediary metabolites, different ligands, and the extent, structure, and biochemistry of the pathway are unclear. Moreover it is unknown whether these sterols have other physiologic functions. Such knowledge could provide novel targets for manipulation of longevity pathways or for combating parasitic diseases. Additionally, understanding the metabolism of cholesterol to bile acid synthetic pathways may yield important insights into cardiovascular disease and obesity. Here we elucidate new components of DA synthesis, including a novel conserved 3-hydroxysteroid dehydrogenase, which plays a key role in the control of reproductive development and longevity. Our findings suggest remarkable conservation of bile acid synthetic pathways, which may have implications for the physiologic role of cholesterol and bile acid homeostasis in higher organisms.

## Results

### Genetic Screens Identify New Components of Dafachronic Acid Biosynthesis

Hormone biosynthetic mutants that reduce production of the dafachronic acids (DAs) have a characteristic phenotypic profile. Partial reduction of the pathway, as seen in *daf-9(rh50)* hypomorphs, results in gonadal migration defects (Mig), in which gonadal distal tip cells fail their scheduled turns and migrate instead into head and tail along the ventral body wall [25]. Strong reduction of the pathway, as seen in *daf-9(dh6)* null mutants, results in 100% penetrant constitutive dauer entry (Daf-c) at all temperatures, with the formation of “partial dauer” larvae, which have the characteristic dauer cuticle but show incomplete radial constriction of the pharynx and body. By contrast, *daf-36(k114)* null mutants display these phenotypes with partial penetrance and only in combination with additional stresses: animals are Mig upon cholesterol deprivation and Daf-c at the elevated temperature of 27°C, phenotypes that are rescued by DA supplementation [24]. These results suggest that DAF-36 may work in a branched pathway in concert with other unknown activities, which ultimately converge on DAF-9 for DA production.

**Figure 1:**
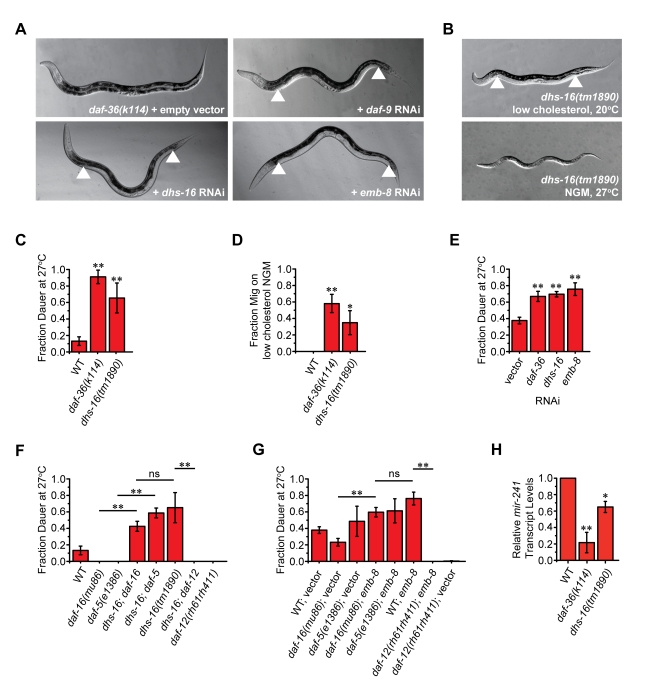
Newly identified loci display phenotypes resembling DA deficiency. (A) Enhancement of *daf-36(k114)* gonadal Mig defects is seen upon knockdown of *daf-9*, *dhs-16*, and *emb-8*. Arrowheads indicate the arms of the gonad, visible along the body due to failure of proper distal tip cell migration. (B) Mig and Daf-c phenotypes of *dhs-16(tm1890)* deletion mutants, under conditions of cholesterol deprivation (upper image) and 27°C (lower image). (C) Analysis of dauer formation at 27°C on NGM. *dhs-16(tm1890)* mutants show Daf-c phenotypes similar to *daf-36(k114)* (*N* = 3, M±SD; **p<0.01). (D) Analysis of the gonadal Mig defects of *dhs-16* null animals on NGM without added cholesterol (*N* = 3, M ± SD; **p<0.01, *p<0.05). (E) RNAi knockdown of *dhs-16* and *emb-8* in wild-type worms at 27°C induces Daf-c phenotypes, similar to knockdown of *daf-36* (*N*≥4, M ± SD; **p<0.005). (F) Genetic epistasis analysis of *dhs-16(tm1890)* (Daf-c) together with Daf-d mutations in transcription factors of insulin/IGF, TGF-β, and DA signaling show that DHS-16 works downstream of DAF-16/FOXO and DAF-5/SKI, but upstream of DAF-12/NHR (*N* = 3, M ± SD; **p<0.01). (G) Similar genetic epistasis analysis of *emb-8* RNAi-induced dauer phenotypes at 27°C suggests that EMB-8 acts downstream of DAF-16 and upstream of DAF-12 (*N* = 3, M ± SD; **p<0.01). (H) The DAF-12 target gene *mir-241*, a *let-7* related microRNA, shows reduced expression in *dhs-16* mutants under low cholesterol conditions at 25°C (*N* = 3, M ± SD; **p<0.01, *p<0.05).

To identify new activities in DA biosynthesis, we conducted genome-wide RNAi screens looking for enhancers of *daf-36(k114)* at the normally permissive temperature of 25°C. We identified several loci that in combination with *daf-36* gave Mig and Daf-c phenotypes and whose molecular identity suggested a role in DA biosynthesis. As expected, RNAi against *daf-9* enhanced *daf-36* mutant phenotypes (Figure 1A). We also identified *ncr-1*, a worm homolog of the Niemann-Pick C1-like proteins implicated in sterol transport that was previously noted to interact with *daf-36* (unpublished data) [24],[30]. Genes with potentially novel roles in DA synthesis included *dhs-16*, a short-chain dehydrogenase/reductase (SDR), and *emb-8*, a NADPH-Cytochrome P450 oxidoreductase. SDRs comprise a large superfamily that typically carry out oxidation/reduction reactions on a variety of substrates, including sterols, xenobiotics, retinoids, and fatty acids [31]. Of the 84 SDRs present in *C. elegans*, the two closest relatives of DHS-16 are DHS-2 and DHS-20, with ∼37% identity in protein sequences. Nematode orthologs include one in the parasitic nematode *Ascaris suum*, which displays ∼40% identity (Figure S1). In humans, the closest relatives include SDR9C7/SDR-O and HSD17B6, which display 40% and 38% identity to DHS-16, respectively (Figure S1). HSD17B6 is involved primarily in androgen metabolism, whereas SDR9C7/SDR-O is expressed almost exclusively in the liver of mice and humans and reportedly metabolizes retinoids [32],[33].

Cytochrome P450 oxidoreductases are obligate co-factors for many CYP450 oxygenases, catalyzing electron transfer from NADPH/NADH to CYP450 enzymes, including those involved in sterol and bile acid synthesis. In humans, NADPH-CYP450 oxidoreductase is required for the activity of most microsomal CYP450 enzymes [34]. The single ortholog found in *C. elegans*, *emb-8* (46% identity), is essential for early embryonic development [35]. EMB-8 is required for CYP450-mediated enzymatic activities in *C. elegans* [36],[37] and presumably serves as a co-factor for DAF-9/CYP450 in DA production.

### Larval Phenotypes Suggest a Role in DA Production

To investigate whether loss of *dhs-16* leads to characteristic phenotypes associated with DA deficiency, we obtained a putative null allele from the knockout consortium (NBP, Japan). *dhs-16(tm1890)* is a 607 bp deletion removing the first exon, including part of the SDR/NAD(P)-Binding Rossman Fold domain (Figure S2). *dhs-16* mutants appear normal at 20°C but have Daf-c phenotypes at 27°C, forming transient partial dauers with incomplete penetrance (65%±11%) (Figure 1B–D, Table 1). They also exhibit gonadal Mig defects upon cholesterol deprivation (37%±13%). Similar larval phenotypes were visible with *dhs-16* RNAi, confirming the *dhs-16* loss of function phenotype (Figure 1E, Table S1). Consistent with a role in DA production, *dhs-16(tm1890)* phenotypes were enhanced by mutations in other biosynthetic genes, including *daf-36(k114)*/Rieske oxygenase and *hsd-1(mg433)*/3β-hydroxysteroid dehydrogenase null mutants, as well as *daf-9(k182)* hypomorphs (Table 1). Comparable enhancement was also seen with mutants of *ncr-1*/Niemann-Pick C1-like protein. Phenotypic enhancement of *daf-36* null animals suggests that DHS-16 works in a branched rather than linear biosynthetic pathway for DA biosynthesis. Similar to *dhs-16*, knockdown of *emb-8* by RNAi resulted in Daf-c phenotypes at 27°C and strongly enhanced the Mig defects of *daf-9(k182)* hypomorphs, *daf-36(k114)* nulls, as well as *dhs-16(tm1890)* deletion mutants in a *daf-12*-dependent manner (Figure 1E, Table S1), consistent with a role in DA production.

**Table 1:** *dhs-16* epistasis and synergy experiments.

Genotype	Daf-c at 25°C ± SE(%)^a^	*N* b	Daf-c at 27°C ± SE(%)^a^	*N* b	Mig at 20°C ± SE(%) NG-chol^c^	*N* b
WT	0±0	1,792(3)	13±3	1,869(3)	0±0	146(3)
*daf-36(k114)*	0±0	467(2)	91±5	2,849(3)	59±11	136(3)
*dhs-16(tm1890)*	0±0	492(3)	65±11	1,252(3)	37±13	158(3)
*dhEx396(dhs-16::gfp)*	nd		0±0	174(3)	0±0	122(3)
*dhs-16; dhEx396*	nd		1±1	335(3)	0±0	150(3)
*daf-5(e1386)*	nd		0±0	1,136(3)	nd	
*dhs-16; daf-5*	nd		59±3	706(3)	nd	
*daf-16(mu86)*	nd		0±0	1,849(3)	nd	
*dhs-16; daf-16*	nd		42±3	2,390(3)	nd	
*daf-12(rh61rh411)*	nd		0±0	1,223(3)	nd	
*dhs-16; daf-12*	nd		0±0	1,072(3)	nd	
*daf-9(k182)*	0±0^d^	1,222(2)	66±29	909(2)	18±0^e^	68(1)
*daf-36;daf-9*	100±0^d^	356(2)	nd		nd	
*dhs-16; daf-9*	14±9^d^	947(2)	nd		64±0^e^	66(1)
*dhs-16; daf-36*	33±4	968(2)	77±7	224(2)	78±2^e^	50(2)
*daf-2(e1370)*	0±0^d^	370(2)	nd		nd	
*dhs-16;daf-2*	3±3^d^	150(2)	nd		nd	
*hsd-1(mg433)*	0±0	964(2)	0±0	909(2)	nd	
*dhs-16; hsd-1*	68±21	628(2)	95±3	565(2)	nd	
*ncr-1(nr2022)*	0±0	660(2)	47±16	342(2)	0±0	78(2)
*dhs-16; ncr-1*	0±0	550(2)	60±3	295(2)	41±7^e^	50(2)

nd, not determined.

a Dauers formed under reproductive growth conditions.

b Number of experiments is given in parentheses.

c Hermaphrodite distal tip cells that fail to turn in L3, *n*>50 cells, grown on NGM without added cholesterol.

d Measured at 20°C.

e Percentage Mig on normal NGM plates with cholesterol.

### Genetic Epistasis Experiments Place *dhs-16* at a Position Consistent with a Role in DA Production

Genetic and molecular experiments reveal that downregulation of insulin/IGF-1 and TGF-β signaling triggers dauer formation by stimulating their respective transcriptional outputs *daf-16*/FOXO, *daf-3*/SMAD, and *daf-5*/SNO-SKI [12]. In addition, significant crosstalk occurs between these pathways. Ultimately, IIS and TGF-β signaling converge upon DA production/DAF-12 activity and are thought to downregulate DA synthesis and promote assembly of a DAF-12/DIN-1 repressor complex that specifies dauer. Loci involved in DA biosynthesis work downstream of IIS and TGF-β signaling components but upstream of DAF-12/NHR, with respect to dauer formation [25],[26]. DA can rescue the Daf-c phenotypes of *daf-2*/InsR, *daf-7*/TGF-β, and *daf-9*/CYP450 mutants, and all DA-related phenotypes show DAF-12 dependence [11]. To determine where DHS-16 acts in these pathways, we conducted epistasis experiments with *dhs-16(tm1890)* Daf-c mutants, constructing strains with the dauer defective (Daf-d) loci *daf-12*/NHR, *daf-16/*FOXO, and *daf-5*/SNO-SKI. Double mutants were then analyzed for whether Daf-c or Daf-d phenotypes prevailed. Whereas *daf-12* completely suppressed *dhs-16* Daf-c phenotypes, *daf-16* and *daf-5* did not (Figure 1F, Table 1). Similarly, Daf-c phenotypes of *emb-8* RNAi induced at 27°C were suppressed by *daf-12* but not by *daf-16* null mutations, placing it at a similar position in the pathway, although unexpectedly *daf-5* mutants formed dauers under RNAi culture conditions (Figure 1G, Table S1). These results place DHS-16, and possibly EMB-8, downstream of DAF-16/FOXO and DAF-5/SKI but upstream of DAF-12/NHR, consistent with a role in DA biosynthesis. Accordingly, expression levels of the *let-7* related microRNA *mir-241*, a direct target gene responsive to DA and DAF-12 in developmental timing events, were down in *dhs-16* mutants (Figure 1H).

### Sterol Supplementation Experiments Position DHS-16 in the DA Biosynthetic Pathway

The nature and intermediates of the DA biosynthetic pathway are not well understood and elements of a pathway have been assembled from fragmentary evidence. Major identified sterols metabolized from cholesterol include 7-dehydrocholesterol and lathosterol, but their function and the enzymes that produce them are unclear. Recently we showed that DAF-36/Rieske oxygenase is involved in the conversion of cholesterol to 7-dehydrocholesterol, revealing this metabolite as a precursor in DA biosynthesis [29]. Best understood is DAF-9/CYP450, with an established activity as a sterol-27-hydroxylase that successively oxidizes the side chain of the 3-keto steroids, lathosterone and 4-cholesten-3-one, to the carboxylic acid moieties of Δ^7^-DA and Δ^4^-DA, respectively [11]. We used these and related sterols to a priori build a pathway model, using metabolic genetic and biochemical approaches.

**Figure 2:**
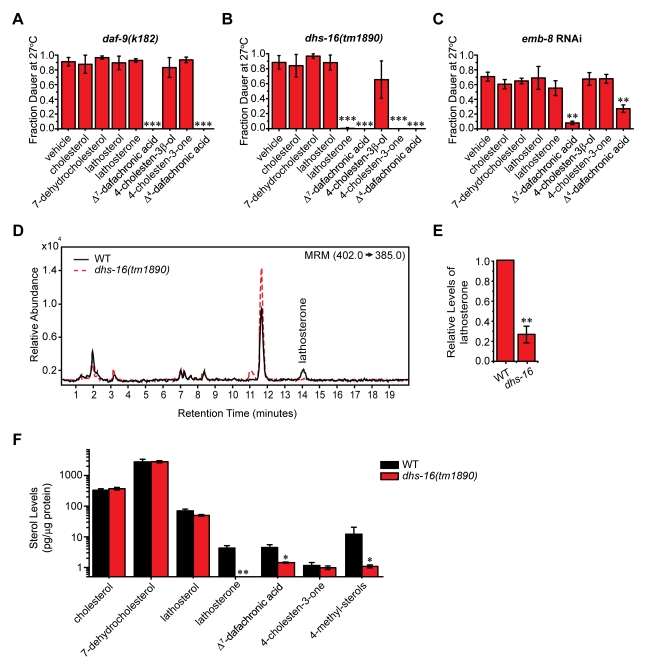
*dhs-16* mutant animals are deficient in lathosterone. (A) *daf-9(k182)* rescue is seen only with the DAs. Experiments were carried out at 27°C with 33 µM concentration of supplemented compounds (*N*≥3, M ± SD; ***p<0.0001). (B) *dhs-16(tm1890)* rescue is seen with lathosterone and dafachronic acids, but not by cholesterol, 7-dehydrocholesterol, and lathosterol. Similarly, 4-cholesten-3-one rescues, but not cholesterol or 4-cholesten-3β-ol. This indicates that *dhs-16* may function in the conversion of lathosterol to lathosterone in the production of Δ^7^-DA and in the formation of 4-cholestene-3-one in the production of Δ^4^-DA (N≥3, M ± SD; ***p<0.0001). (C) *emb-8* RNAi rescue is seen only with the DAs, and not with lathosterone or 4-cholesten-3-one. (*N*≥3, M ± SD; **p<0.01). (D) LC/MS/MS analysis of lipid extracts from L3 stage animals reveals that lathosterone levels are reduced in *dhs-16(tm1890)* mutant animals compared to N2 wild-type (WT) animals, shown quantitatively in (E). Lathosterone levels are significantly reduced in *dhs-16* animals relative to N2 wild-type (3.5-fold decrease; *N* = 7, M ± SD; **p<0.001). (F) GC/MS/MS analysis of sterol levels in *dhs-16* mutants reveals deficiencies in lathosterone, Δ^7^-dafachronic acid, and 4-methyl sterols compared to N2 wild-type (*N*≥10, M ± SEM; *p<0.05, **below detection limit).

To place *dhs-16* at a specific step in DA biosynthesis, we first performed sterol supplementation experiments by feeding loss-of-function mutants proposed DA precursors, including cholesterol, lathosterol, lathosterone, and 4-cholesten-3-one, and looked for rescue of Daf-c phenotypes at 27°C. Compounds working downstream or parallel to the synthetic block should rescue mutant phenotypes, while those working upstream should not. Consistent with working at the last step, the *daf-9(k182)* hypomorphic mutant was fully rescued only by the DAs (Figure 2A), while the Daf-c ligand-insensitive *daf-12(rh273)* mutant was mostly unaffected by provision of DAs (Figure S3A). In accord with a role in DA production, *dhs-16* mutants were rescued by Δ^4^-DA and Δ^7^-DA (Figure 2B).

Strikingly, *dhs-16* mutants were also rescued by the 3-keto steroid lathosterone, the proposed precursor of Δ^7^-DA, but not by the 3β-hydroxyl steroid lathosterol, nor by cholesterol and 7-dehydrocholesterol (Figure 2B). Similarly, rescue was seen with the 3-keto steroid 4-cholesten-3-one, the proposed precursor of Δ^4^-DA, but not by 4-cholesten-3β-ol. These results indicate that the 3-alcohol sterols (cholesterol, 7-dehydrocholesterol, lathosterol, and 4-cholesten-3β-ol) lie upstream of the block, whereas the 3-keto steroids (lathosterone, 4-cholestene-3-one, and the DAs) lie downstream. Furthermore, lathosterone rescued animals at nanomolar concentrations more efficiently than 4-cholesten-3-one (Figure S3C). This may be due to the 5-fold lower activity of Δ^4^-DA relative to Δ^7^-DA, rendering precursors of Δ^4^-DA less able to rescue [38]. Alternatively, DHS-16 may act with greater specificity in the Δ^7^-DA branch. Also consistent with a role downstream of *daf-36*, the Daf-c phenotype of *dhs-16;daf-36* double mutants was rescued by provision of lathosterone and the dafachronic acids in a manner similar to *dhs-16* single mutants (Figure S3C–E). Given the structures of these molecules, the simplest hypothesis is that DHS-16 converts 3-hydroxy steroids to 3-keto steroids—that is, it functions as a 3β-hydroxysteroid dehydrogenase in the production of the DAs. If so, DHS-16 is surprisingly not orthologous in sequence to known 3β-hydroxysteroid dehydrogenase enzymes, thereby identifying a novel function for a conserved orphan dehydrogenase.

We also examined rescue of *emb-8* RNAi Daf-c phenotypes by proposed DA precursors and found that rescue was achieved only by provision of the DAs and not by lathosterone or 4-cholesten-3-one (Figure 2C), suggesting *emb-8* functions at the same step as *daf-9*, consistent with a role as a CYP450 reductase.

### DHS-16 Has Lathosterol 3β-Dehydrogenase Activity

The feeding experiments described above indicated that DHS-16 may act in the production of lathosterone and possibly 4-cholesten-3-one. To further investigate this hypothesis, synchronized wild-type N2 and *dhs-16* mutant animals were cultured in liquid media *en masse* and harvested at the L3 stage. Lipid extracts were obtained from these animals and analyzed by LC/MS/MS for changes in proposed DA precursors. Consistent with predictions based on feeding experiments, *dhs-16* mutants had 3.5-fold less lathosterone compared to wild-type animals (*p*<0.0001), indicating that *dhs-16* is required for production of lathosterone (Figure 2D–E).

Similar results were also obtained by growing worms on solid media and analyzing lipid extracts by GC/MS/MS: *dhs-16* mutants had levels of the putative product lathosterone that were below the limit of detection, as well as significantly decreased levels of the downstream product Δ^7^-DA compared to wild-type (1.5 versus 4.5 pg/µg protein, *p*<0.05) (Figure 2F). The residual Δ^7^-DA seen in *dhs-16* mutants suggests that other pathways exist for its production. Δ^4^-DA was below the detection limit for both WT and mutant extracts (unpublished data). Surprisingly, levels of the putative precursor lathosterol did not accumulate, nor did cholesterol, 7-dehydrocholesterol, or 4-cholesten-3-one (Figure 2F). These results suggest the pathway is not strictly linear and that lathosterol may be metabolized through other pathways. Interestingly, *dhs-16* mutants also showed significantly decreased levels of 4-methyl sterols (consisting of lophenol and 4-methyl-5α-cholest-8(14)-en-3β-ol), suggesting a role in their production (Figure 2F). 4-methyl derivatives are nematode-specific modifications catalyzed by STRM-1 methyltransferase. STRM-1 is proposed to irreversibly shunt sterols, such as lathosterone and 4-cholestene-3-one, away from DA production and thereby regulate dauer formation [39]. Taken together, these results reveal that DHS-16 is required for the normal production of lathosterone, which subsequently impacts levels of Δ^7^-DA, and that earlier proposed intermediates may have complex branch points or tissue-specific changes.

**Figure 3:**
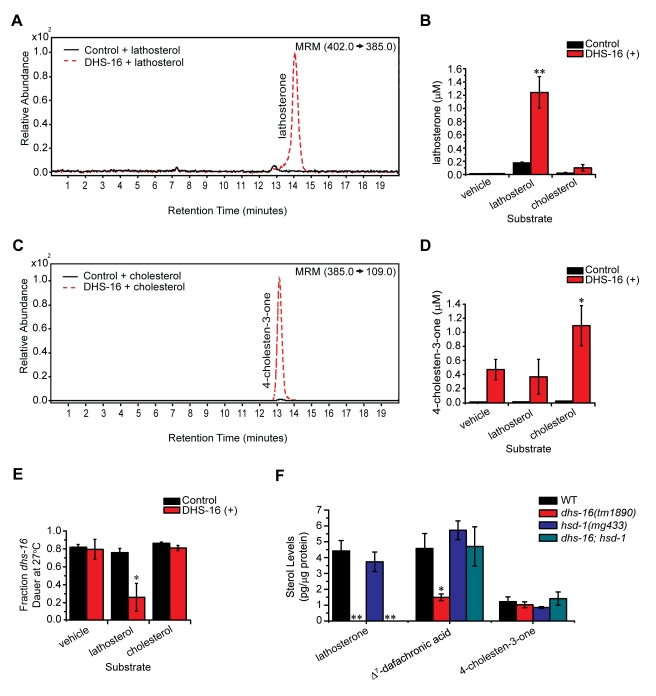
DHS-16 acts as a 3-hydroxysteroid dehydrogenase in DA biosynthesis. (A) LC/MS/MS analysis of lipid extracts from DHS-16 and control microsomes incubated with the proposed substrate lathosterol, shown quantitatively in (B). Significantly more lathosterone is detected in incubations of DHS-16 microsomes with lathosterol than in incubations with empty vehicle ethanol controls. DHS-16 microsomes also do not produce lathosterone when incubated with cholesterol, demonstrating that specific products are made depending upon the substrate provided (*N*≥3, M ± SD; **p<0.005). (C) LC/MS/MS analysis of 4-cholesten-3-one levels in lipid extracts from DHS-16 and control microsomes incubated with cholesterol, shown quantitatively in (D). Concentrations of 4-cholesten-3-one in incubations of DHS-16 microsomes with cholesterol are significantly greater than that seen in control microsomes or incubations with lathosterol (*N* = 3, M ± SD; *p<0.05). (E) Rescue of the Daf-c phenotype of *dhs-16(tm1890)* mutants at 27°C is seen when fed lipid extracts of DHS-16 microsomes incubated with the proposed substrate lathosterol. Rescue is not seen with extracts from DHS-16 microsomes incubated with ethanol vehicle alone or with cholesterol, and extracts from empty vector pCMV control microsomes do not rescue in any condition (estimated concentrations of 300 nM in plates; *N* = 3, M ± SEM; *p<0.05). (F) GC/MS/MS analysis of sterol levels in N2 wild-type, *dhs-16(tm1890)*, *hsd-1(mg433)*, and *dhs-16;hsd-1* double mutants reveals that *hsd-1* is not required for 4-cholesten-3-one production, as previously proposed, suggesting HSD-1 may act in an alternative parallel pathway. In addition, although the *dhs-16;hsd-1* double mutants did not contain measurable lathosterone levels, Δ^7^-DA levels were not reduced, suggesting that lathosterone is not required for its production and that alternate pathways must exist that are independent of *dhs-16* (*N*≥6, M ± SEM; **below detection limit, *p<0.05).

In order to directly test whether DHS-16 could convert 3-hydroxysteroids to 3-ketosteroids, we expressed FLAG-tagged DHS-16 in HEK293T cells and incubated microsomes isolated from these cells with putative substrates. LC/MS/MS analysis of lipid extracts from these incubations revealed a dramatic increase in lathosterone concentration when DHS-16(+) microsomes were incubated with the substrate lathosterol compared to controls (*p*<0.005) (Figure 3A–B). In addition, DHS-16(+) microsomes were also capable of producing 4-cholesten-3-one when incubated with cholesterol, as levels of this compound were produced at significantly higher concentrations compared to controls (*p*<0.05) (Figure 3C–D). When extracts from these incubations were fed to *dhs-16* mutants, only incubations with lathosterol were able to rescue the Daf-c phenotypes of these animals at 27°C, whereas extracts from incubations with the vehicle control ethanol or cholesterol could not, consistent with the decreased ability of 4-cholesten-3-one to rescue at low concentrations (Figure 3E). In sum, these biochemical data demonstrate that DHS-16 has activity similar to 3β-hydroxysteroid dehydrogenases in the conversion of 3-alcohol to 3-keto steroids.

Although DHS-16 is capable of producing of 4-cholesten-3-one in vitro, analysis of worm extracts suggested that it is not required for this activity (Figure 2F, Figure 3F). Another enzyme, the canonical 3β-hydroxysteroid dehydrogenase HSD-1, has been postulated to carry out this reaction [27], and therefore could work in parallel to DHS-16. Accordingly, *dhs-16* mutants synergized strongly with *hsd-1* (61% Daf-c, 25°C), more so than with *daf-36* (30% Daf-c, 25°C), despite the fact that *dhs-16* and *hsd-1* single mutants had weaker phenotypes than *daf-36* (Table 1). Sterol analysis of *hsd-1* single mutants, however, revealed no significant reduction in levels of 4-cholesten-3-one and Δ^7^-DA at 20°C (Figure 3F, Figure S4A). In addition, at the elevated temperature of 25°C, these animals displayed elevated levels of Δ^7^-DA relative to wild-type (Figure S4B). Thus, while HSD-1 may act in a parallel pathway, it is not required for 4-cholesten-3-one nor Δ^7^-DA production. Moreover, the sterol rescue profile of *dhs-16;hsd-1* double mutants resembled that of *dhs-16* alone, exhibiting rescue of Daf-c phenotypes at 27°C by excess lathosterone, 4-cholesten-3-one, and Δ^7^-DA (Figure S3F). Also similar to *dhs-16* single mutants, *dhs-16;hsd-1* double mutants were deficient in lathosterone. At 20°C, double mutants showed no decrease in 4-cholesten-3-one levels, and surprisingly no change in Δ^7^-DA levels, suggesting that *hsd-1* reverses the Δ^7^-DA deficiency seen in *dhs-16* single mutants at this temperature. By contrast, at 25°C, Δ^7^-DA levels were lower in double mutants (Figure S4B), consistent with the stronger phenotypes of these animals at elevated temperatures. Taken together these observations suggest that other pathways must exist for Δ^7^-DA production independent of lathosterone and *dhs-16* and that additional dafachronic acid ligands or inhibitors may also exist, which may interact with complex regulation.

### Hypodermal DAF-9 Expression Reflects Decreased Dafachronic Acid Levels in *dhs-16* Mutants

To further elucidate the activity of DHS-16 in the DA pathway we examined its behavior in hormonal feedback loops. Previous studies demonstrated that *daf-9* expression in the hypodermis is an important point of control. Hypodermal *daf-9::gfp* exhibits low levels of expression in wild-type animals under normal growth conditions, but is shut off in response to stressful dauer-inducing conditions during the L2 stage [40],[41]. By contrast, it is upregulated in response to weak stress, including elevated temperatures (25°C), reduced food or cholesterol, and modest exposure to dauer pheromone, in animals undergoing reproductive growth. This upregulation is also seen in normal growth conditions in mutants that partly diminish IIS, TGF-β signaling, and DA biosynthesis, suggesting these pathways convey environmental stress and converge on regulation of *daf-9*. Hypodermal *daf-9::gfp* shows strict dependence on *daf-12*, as expression is absent in *daf-12* null mutants but enhanced in ligand insensitive mutants, suggesting both positive regulation and negative feedback control by DAF-12 [40],[41].

**Figure 4:**
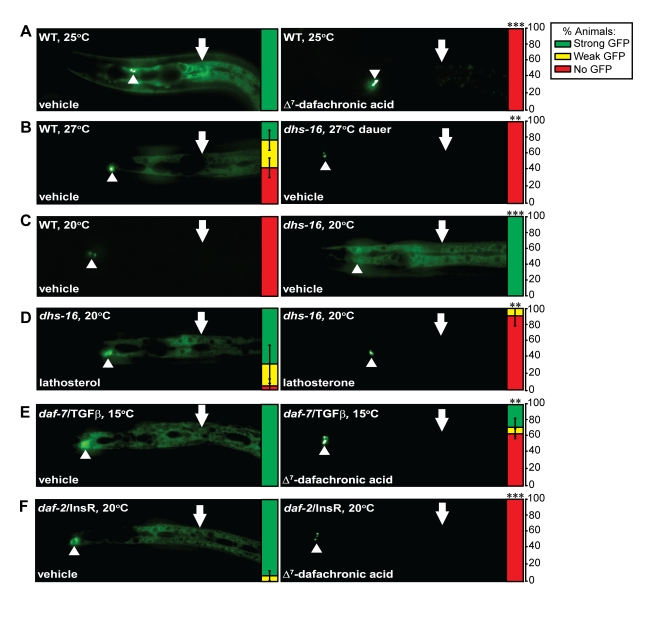
*dhs-16* modulates feedback regulation of hypodermal *daf-9* expression. (A) In response to mild stress (e.g., growth at 25°C), hypodermal *daf-9* is upregulated in N2 wild-type animals (WT) carrying an integrated *dhIs64(daf-9::gfp)* array (shown at left). This is rescued upon growth with 33 µM Δ^7^-DA (shown at right). Arrowheads indicate the XXX R/L neuroendocrine cells in which *daf-9* expression is relatively unchanged, and arrows indicate hypodermal expression. The expression levels are displayed quantitatively to the right of each image, as the percentage of animals observed with strong (green), weak (yellow), or no (red) hypodermal GFP expression (*N*≥3, M ± SD; ***p<0.0001). (B) Under stressful growth conditions at 27°C N2 wild-type animals still undergo reproductive development but have high hypodermal *daf-9::gfp* expression (left), whereas *dhs-16* mutants mostly develop as dauer larvae and shut off hypodermal *daf-9::gfp* expression (right) (**p<0.01). (C) Under normal growth conditions at 20°C, hypodermal *daf-9* upregulation is low in wild-type (left), whereas upregulation is seen in the *dhs-16(tm1890)* mutant background (right), suggesting *daf-9* upregulation in response to DA deficiency (***p<0.0001). (D) Upregulation of hypodermal *daf-9::gfp* seen in *dhs-16* mutants is not rescued by provision of 33 µM of the upstream DA precursor lathosterol (left) but is rescued by the downstream product lathosterone (right) (**p<0.01). (E) Upregulation of hypodermal *daf-9::gfp* seen in reproductively growing *daf-7(e1372)*/TGF-β mutants (left) is rescued by DA (right) (**p<0.01). (F) Upregulation of hypodermal *daf-9::gfp* seen in reproductively growing *daf-2*/InsR mutants (left) is also rescued by DA (right) (***p<0.0001).

We wondered whether the environmental and genetic effects on hypodermal *daf-9::gfp* expression ultimately reflect DA/DAF-12 signaling. If so, then hypodermal *daf-9* expression should be responsive to DA. We found that under conditions of mild thermal stress (25°C, 27°C) upregulation of hypodermal *daf-9::gfp* in wild-type animals was reversed by excess Δ^4^- or Δ^7^-DA (Figure 4A, Figure S5), providing direct evidence that the effect of temperature on *daf-9::gfp* expression reflects DA availability. We next examined the regulatory behavior in response to perturbations in DA biosynthesis. When grown at 27°C, *dhs-16* mutants either entered the dauer diapause and shut off hypodermal *daf-9::gfp* (Figure 4B) or bypassed dauer and massively upregulated expression (Figure S5). When grown in normal 20°C growth conditions, *dhs-16* mutants stimulated high expression of hypodermal *daf-9::gfp* relative to wild type (Figure 4C). The high expression of *daf-9::gfp* is consistent with reduced DA levels, since its expression was strikingly reduced to wild-type levels upon feeding lathosterone as well as the DAs, but not lathosterol (Figure 4D, Figure S5). By contrast, 4-cholesten-3-one failed to rescue the upregulation seen in *dhs-16* mutants, again suggesting that *dhs-16* function may be more crucial to the Δ^7^-DA branch (Figure S5). Finally, we found that *daf-9::gfp* upregulation seen in *daf-7*/TGF-β and *daf-2*/InsR mutants at 15°C and 20°C was also reversed by Δ^7^-DA (Figure 4E–F). Altogether these data suggest that IIS, TGF-β, and hormone biosynthesis normally promote DA signaling, which feeds back on hypodermal *daf-9* (see Discussion for model).

### DHS-16 Is Expressed in Multiple Endocrine Tissues and Is Regulated by Insulin/IGF-I Signaling

**Figure 5:**
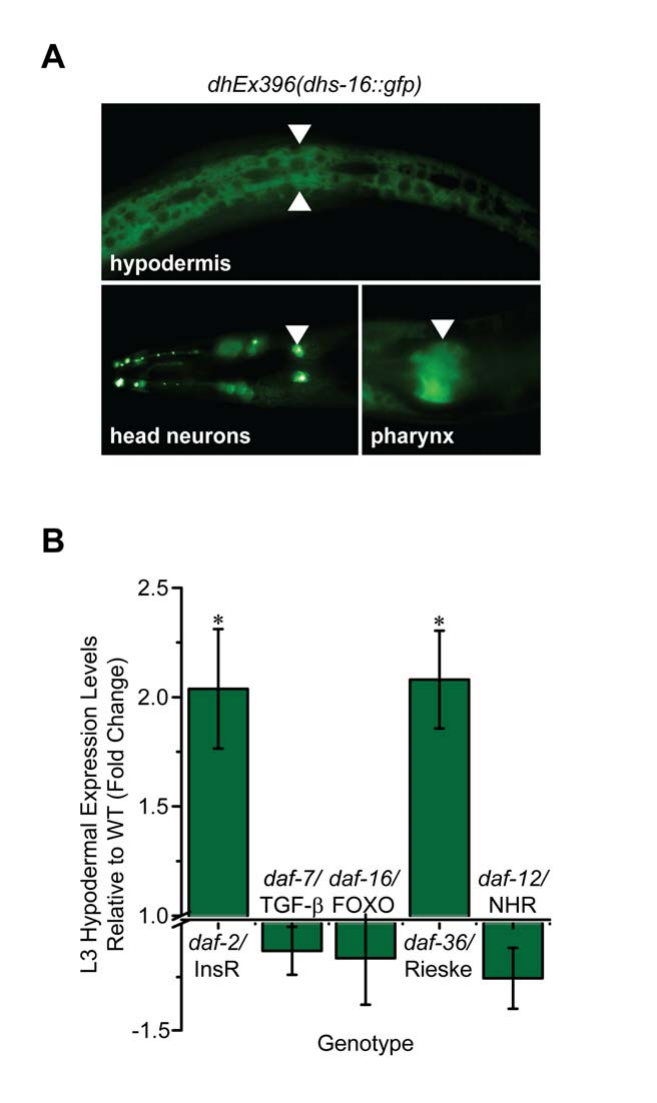
*dhs-16* expression pattern and regulation. (A) A functional *dhs-16::gfp* is expressed in the hypodermis (arrowheads), head neurons (arrows), and posterior pharyngeal bulb (arrowheads). (B) Reduction of IIS in the *daf-2(e1368)* background results in 2-fold upregulation of *dhs-16::gfp* in the hypodermis of L3 stage animals at 20°C (*p<0.05), although no significant change was seen in the absence of *daf-16*/FOXO, *daf-12*/NHR, or *daf-7*/TGFβ. Similar upregulation was seen in the *daf-36(k114)*/Rieske oxygenase mutant background, consistent with a role of *dhs-16* downstream of *daf-36* in DA production (*N*≥3, M ± SEM, *p<0.05).

To gain insight into DA producing endocrine tissues and regulation, we established transgenic lines carrying *dhs-16::gfp* extrachromosomal arrays and analyzed the expression pattern. These C-terminal *gfp* fusions were functional as measured by efficient rescue of both Daf-c and Mig phenotypes of *dhs-16(tm1890)* mutants (Table 1). *dhs-16::gfp* was strongly expressed in the hypodermis, and thus partially overlaps with *daf-9* expression. It was also expressed robustly in the posterior pharyngeal bulb, which is involved in feeding/pumping behavior and concentrates sterols [42], as well as a handful of unidentified head and tail neurons (Figure 5A)—that is, in cells not overlapping with *daf-9* [40]. When crossed into the background of *daf-2*/InsR mutants, we observed a 2-fold upregulation of *dhs-16::gfp* in the hypodermis of L3 stage animals as well as in dauer larvae (*p*<0.05) (Figure 5B, unpublished data). By contrast, no significant changes were observed in the absence of *daf-16*/FOXO, *daf-12*/NHR, or *daf-7*/TGF-β. Similar upregulation was seen in the absence of *daf-36*/Rieske oxygenase (Figure 5B), consistent with a role downstream of *daf-36* in DA biosynthesis and responsive to perturbations in the pathway.

### *dhs-16* Is Partially Required for Longevity in the Absence of the Germline

**Figure 6:**
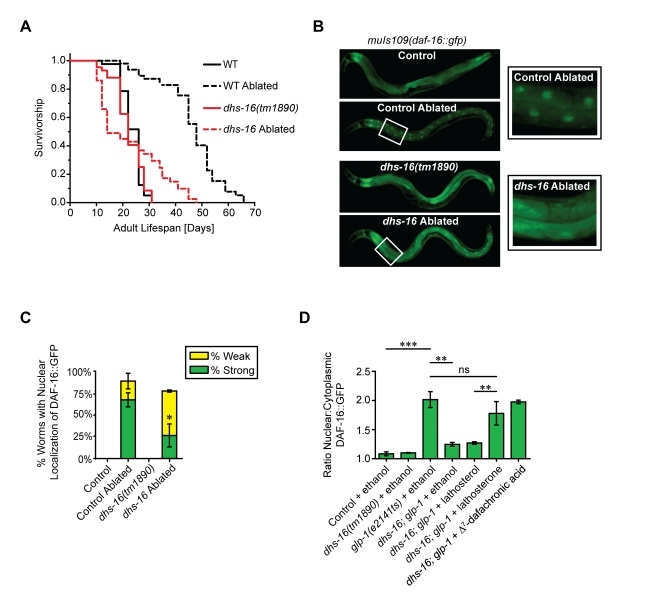
*dhs-16* is partially required for longevity in the absence of the germline. (A) Lifespan of *dhs-16(tm1890)* animals after ablation of germline precursor cells by laser microsurgery. One representative experiment is shown. N2 wild-type (WT) animals live twice as long when the germline is ablated. Longevity is significantly attenuated in *dhs-16* ablated animals (*N* = 2, *p*<0.0001). (B) DAF-16::GFP strongly localizes to intestinal nuclei of day 1 adults after ablation of the germline by laser microsurgery in control animals, whereas the degree of localization is reduced in germline-ablated *dhs-16* mutants. Magnified views of boxed regions are shown to the right. (C) The degree of DAF-16::GFP nuclear localization after germline ablation is shown quantitatively as the percentage of animals with strong (green) or weak (yellow) localization (*N* = 3, M ± SD; *p<0.05). (D) The ratio of intestinal DAF-16::GFP intensity in the nucleus versus cytoplasm reveals increased levels of nuclear expression in germline deficient *glp-1(e2141ts)* mutants at the restrictive temperature of 25°C compared to control animals. Localization is significantly decreased in *dhs-16;glp-1* double mutant animals, and is restored upon provision of lathosterone or Δ^7^-dafachronic acid, but not lathosterol (*N* = 3, M ± SD; ***p<0.0001, **p<0.001).

In the gonadal longevity pathway, animals lacking germline stem cells live 50%–60% longer than animals with an intact gonad [15],[43]. Increased lifespan is not due to sterility as animals lacking both somatic gonad and germline have normal lifespans. It is thought that gonadal signals act in an opposing manner, with the germline producing lifespan-shortening and somatic gonad producing lifespan-extending signals. The gonadal longevity pathway is dependent upon *daf-16*/FOXO and *daf-12*/NHR, as well as the hormone biosynthetic genes *daf-36*/Rieske oxygenase and *daf-9*/CYP450 [15],[25],[44]. Ablation of the germline precursors by laser microsurgery leads to an increased lifespan in wild-type animals, which is no longer seen in these mutant backgrounds. Recently, lifespan-extending signals from the somatic gonad were suggested to be the DAs themselves, because *daf-9* is expressed in the spermatheca and the short lifespan of somatic gonad-ablated animals is restored by feeding DAs [45]. Given the importance of DAs in this process, we asked whether *dhs-16* also functions in the gonadal longevity pathway. We found that the lifespan of *dhs-16* null mutants after removal of the germline precursors by laser microsurgery was significantly reduced (mean = 19±3 d) compared to that of wild-type (mean = 41±3 d; *p*<0.0001) (Figure 6A, Table S2). The failure of a complete suppression of longevity in these animals may be due to residual DA production. Interestingly, aging populations of germline-ablated *dhs-16* mutant animals showed early short-lived but later long-lived mortality trajectories, with an inflection point around days 10–12 seen in both independent experiments, suggesting two subpopulations or distinct temporal events.

A molecular correlate of gonadal longevity is the localization of GFP-tagged DAF-16 to intestinal nuclei, dependent upon *daf-12*, *daf-36*, and *daf-9* [17],[46],[47]. We performed laser microsurgery on animals carrying an integrated *daf-16*::*gfp* and found that DAF-16 nuclear localization was much weaker in *dhs-16* mutants compared to wild-type (*p*<0.05) (Figure 6B–C). As seen in the lifespan studies, we also noted two distinct populations in DAF-16 localization, weakly versus strongly nuclear-localized. However, no correlation was found between strong localization and longevity (unpublished data). Thus, DHS-16 functions in the gonadal longevity pathway, presumably due to a requirement for maximal DA production to promote long life in the absence of the germline.

To see if *dhs-16* phenotypes in the gonadal pathway stem from a deficiency in DA production, we asked whether DAF-16::GFP localization defects could be rescued by lathosterone and DA. To do this, we used a genetic model of germline ablation *glp-1(e2141ts)* and measured the ratio of nuclear to cytoplasmic DAF-16::GFP expression in individual intestinal cells. As seen in the laser ablation experiments, *glp-1* induced DAF-16::GFP nuclear localization in the intestine during the first day of adulthood, which was significantly reduced in the *dhs-16;glp-1* double mutant background (*p*<0.001) (Figure 6D). Similar results were obtained upon treatment of *glp-1* animals with *dhs-16* RNAi (Figure S6). Supplementation with DA or lathosterone, but not lathosterol, restored DAF-16::GFP nuclear localization in both cases. These results suggest that DHS-16 normally promotes DA signaling, which stimulates DAF-16/FOXO nuclear localization in animals lacking the germline. Thus, *dhs-16* contributes to gonadal longevity by linking DA production to DAF-12 activity and nuclear localization of DAF-16/FOXO.

## Discussion

Endogenous small molecule metabolites that regulate animal longevity are emerging as a novel approach to influence health and life span. The dafachronic acids are an important class of bile acid metabolites that regulate *C. elegans* longevity through DAF-12 nuclear receptor signal transduction. DA signaling has also been implicated in the control of nematode parasitism. Here we have identified new components involved in DA biosynthesis, including DHS-16, a short chain dehydrogenase, and EMB-8, a cytochrome P450 reductase, and deduced the biochemical activity of DHS-16 to be a novel 3β-hydroxysteroid dehydrogenase. By controlling ligand availability, DHS-16 regulates DAF-12 activity and thereby directly influences the life plan and life span of *C. elegans*. Importantly, identified intermediates in bile acid metabolism may also provide unique ways to impact both animal longevity and nematode pathogenesis.

Several lines of evidence reveal that DHS-16 is a DA hormone biosynthetic gene. First, mutant animals show the phenotypic profile of other DA pathway mutants, including Daf-c and gonadal Mig phenotypes, perturbation in *daf-9* feedback regulation, and abrogation of life span extension when the germline is absent. Second, mutants have a similar pattern of genetic epistasis, working downstream of insulin/IGF and TGF-β signaling but upstream of *daf-12*, and enhancing phenotypes of other mutations in DA biosynthetic genes. Third, mutant phenotypes are rescued by DA as well as specific precursors in the biosynthetic pathway. Fourth, mutants have decreased expression of a DAF-12/DA dependent target gene *mir-241*. Partial loss of *emb-8* function also shares a similar spectrum of larval phenotypes.

Our data support a role of DHS-16 in the 3β-dehydrogenation of lathosterol to lathosterone, which is required for maximal biosynthesis of Δ^7^-DA. Feeding experiments predict a defect in the conversion of lathosterol to lathosterone, suggesting a function in the metabolism of 3-alcohol to 3-keto steroids. In concert with this, sterol profiles reveal that mutants are deficient in the putative product lathosterone as measured by mass spectrometric methods. Although *dhs-16* mutants do not accumulate the putative precursor lathosterol, this outcome might be expected if lathosterol can be metabolized through other pathways. Also because absolute levels of lathosterol are an order of magnitude greater than lathosterone, perturbation of this step might have a negligible effect. Importantly, microsomes expressing DHS-16 are able to convert lathosterol to lathosterone, critical in vitro evidence that DHS-16 possesses or supports this biochemical activity. DHS-16 is also capable of producing 4-cholesten-3-one from cholesterol in vitro, although *dhs-16* mutants still contain normal levels of this sterol. It seems likely, however, that DHS-16 has other substrates and activities. In the future, it should be interesting to determine the activities of the parasitic nematode DHS-16 orthologs, as they could be important therapeutic targets for treating such pathogens.

**Figure 7:**
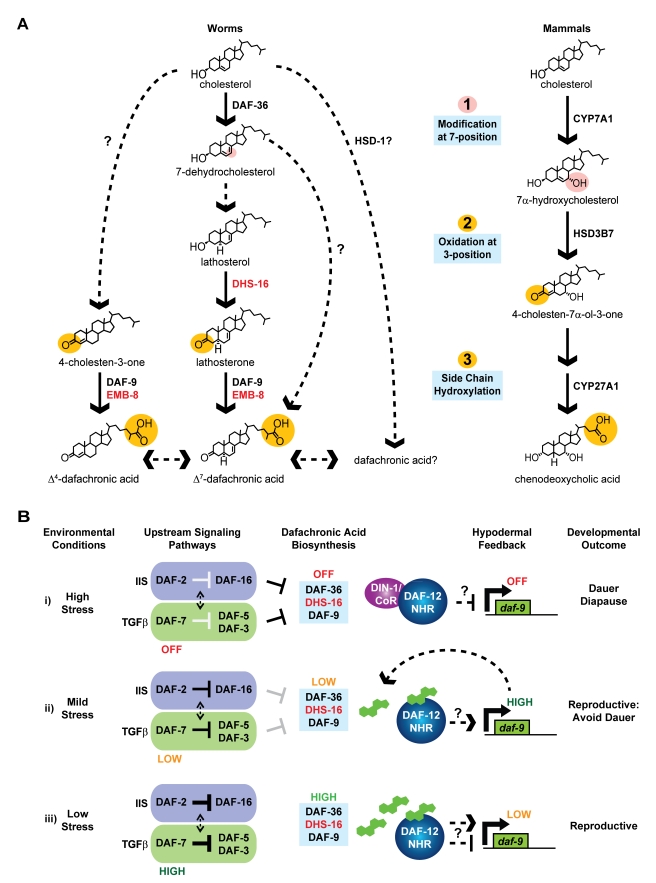
Biosynthesis and regulation of nematode bile acids. (A) A revised model of the dafachronic acid biosynthetic pathway from dietary cholesterol, with newly identified activities shown in red. Although *dhs-16* is required for lathosterone production, mutant animals still produce low levels of Δ^7^-DA. An alternative pathway for Δ^7^-DA synthesis is therefore likely. In addition, *hsd-1* is not required for 4-cholesten-3-one production as previously proposed, but may be involved in producing alternative dafachronic acids. These ligands may have complex regulation and influence the synthesis of one another. Comparison to mammalian bile acid synthesis (right) reveals conserved aspects of bile acid biochemistry. Nematode and mammalian bile acid synthesis involves modification at the 7-position (shown in pink), which speculatively may partition cholesterol towards bile acid synthesis, and in both pathways oxidation of the 3-alchohol and oxidation of the sidechain at the 27-position occurs (shown in orange). (B) Model of hormonal feedback on hypodermal *daf-9::gfp* expression. (i) Stressful environmental conditions result in downregulation of IIS and TGF-β signaling, suppression of DA synthesis, and hypodermal *daf-9* expression by the DAF-12/DIN-1 repressor complex, resulting in dauer formation. (ii) Moderately stressful environments result in modest downregulation of dauer signaling pathways and DA synthesis, with compensatory upregulation of hypodermal *daf-9*, allowing for reproductive development. (iii) In favorable environments with low levels of stress, active IIS and TGF-β signaling results in ample DA production, with low expression of hypodermal *daf-9*. Note it is unknown whether DAF-12 regulates *daf-9* directly or indirectly.

By elucidating metabolites and associated biochemical activities, the overall architecture of the *C. elegans* DA biosynthetic pathway is emerging, revealing not only key changes to previously proposed models, but also that critical biochemical aspects of metazoan bile acid metabolism are remarkably conserved (Figure 7A). The first step in the Δ^7^-DA branch, the conversion of cholesterol to 7-dehydrocholesterol, is carried out by the DAF-36/Rieske oxygenase [24],[29]. A similar activity is proposed for the *Drosophila* homolog, *neverland*, in ecdysteroid biosynthesis [48],[49]; thus, elucidation of early steps in the nematode pathway could illuminate other unsolved analogous early steps in ecdysteroid biosynthesis. Although mammals lack an obvious homolog of this Rieske oxygenase, the first step in mammalian bile acid biosynthesis involves 7-hydroxylation of cholesterol by CYP7A1 [24],[50]. Speculatively, this may indicate that chemical modification at the 7-position of the sterol nucleus is important for partitioning cholesterol towards bile acid synthesis. 7-dehydrocholesterol may then be converted to lathosterol by an as yet unidentified 5α-reductase, as in mammalian pathways. Although *C. elegans* harbors multiple 5α-reductase homologs, RNAi knockdown did not display phenotypes typical of DA deficiency (Wollam and Antebi, unpublished), possibly because of redundancy. DHS-16 is implicated as a 3β-hydroxysteroid dehydrogenase in the conversion of lathosterol to lathosterone. A similar activity, albeit on different substrates, is ascribed to the mammalian 3β-HSDs (e.g., HSD3B7) [50], yet DHS-16 is not orthologous to these enzymes, and uniquely may not involve obligate Δ^4^/Δ^5^-isomerization for activity. We suggest that the discovery of DHS-16 activity in worms might inform mammalian biology, and we speculate that the liver-expressed SDR-O/SDR9C7 could have an analogous activity in mammalian bile acid synthetic pathways. Although *dhs-16* affects Δ^7^-DA levels, additional *dhs-16* and lathosterone-independent mechanisms for the synthesis of Δ^7^-DA appear to exist. Next, a critical step in bile acid synthesis entails the successive oxidation of the cholesterol side chain to the 26-carboxylic acid moiety, which is carried out by DAF-9/CYP450 in a manner similar to mammalian CYP27A1. This step is likely facilitated by EMB-8/CYP450 reductase, since the Daf-c phenotypes of *emb-8* RNAi map to the same step as *daf-9*/CYP450 based on sterol rescue experiments. Finally, our data also suggest that HSD-1 is not required for 4-cholesten-3-one production as previously proposed, or for Δ^7^-DA production under some conditions, but is likely acting in another pathway. In particular, the unexpected observation that loss of *hsd-1* restores Δ^7^-DA production in *dhs-16* mutants at 20°C implies there are additional pathways and ligands with complex regulation that remain to be found. As there are numerous HSD, DHS, and CYP450 enzymes in *C. elegans* with potential to carry out such reactions, examination of double and triple mutants as well as enzymatic activities will be required to resolve these issues in the future.

Homologs of other genes implicated in mammalian bile acid metabolism are found in the *C. elegans* genome, including the D-bifunctional protein and Sterol Carrier Protein-χ, which function in shortening side chains of mammalian bile acids [50]. The *C. elegans* counterparts, DHS-28 and DAF-22, have a different known role, promoting synthesis of dauer pheromone by shortening the long chain fatty acid side chains of ascarosides [51]. Intriguingly, dauer pheromone works in a manner opposite to DA to promote dauer formation, but it is unknown whether DAF-22 and DHS-28 also influence *C. elegans* bile acid metabolism or whether shorter chain bile acids are found in the worm. In conclusion, it is striking that many of the activities in the nematode pathway are biochemically analogous to those found in mammals, yet the enzymes are not always strict structural orthologs, suggesting potential convergent evolution (Figure 7A). Speculatively, this similarity might have been exploited by parasitic nematodes to signal entry into the appropriate host.

Although *daf-9* and *dhs-16* expression partially overlap in the hypodermis, *daf-36* is found in non-overlapping tissues including the intestine as well as epidermal seam cells in dauers [24],[25]. The fact that the various gene products have distinct expression patterns suggests distributed synthesis of DA, which likely requires transport mechanisms. ABC and Niemann-Pick Type C1-like transporters, which are also implicated in dauer formation (*mrp-1, ncr-1,-2*), may be involved [30],[52]. Distributed synthesis could be deployed to make specific DAF-12 ligands with various activities, to localize activity within particular tissues, or to develop a consensus mechanism enabling each tissue to influence the dauer decision.

Regulatory feedback circuitry is a common feature of both mammalian and *C. elegans* bile acid-like synthesis, dependent upon FXRα and its homolog DAF-12, respectively. In mammals, feedback regulation converges on CYP7A1, although hints of CYP27A1 regulation are evident [53]. In *C. elegans*, DA production is regulated primarily through hypodermal *daf-9*/CYP450 expression [40],[41], but other control points, such as *dhs-16* and *daf-36*, are now evident and deserve closer study. Our observations on the regulation of hypodermal *daf-9* expression and its dependence on DA suggests a three-state model in which environmental cues work through dauer signaling pathways and DA biosynthesis to dictate regulation of *daf-9* expression through *daf-12* dependent feedback (Figure 7B): (i) Harsh stress results in a shutdown of DA production, repression of *daf-9* expression, and dauer formation; (ii) mild stress initially results in decreased DA production, provoking compensatory upregulation of *daf-9* to maintain hormone levels and reproductive development; and (iii) favorable conditions result in DA excess causing negative feedback on *daf-9* expression and reproductive development [40]. *dhs-16* regulation is somewhat different. Contrary to predictions from genetic epistasis, *dhs-16::gfp* is not downregulated by decreased IIS or TGF-β, but surprisingly upregulated by reduced IIS and DA. These observations suggest that other regulatory points in the pathway, such as *daf-9*, take precedence, or that regulation takes place at the level of DHS-16 activity or metabolite production. Moreover it also implies that *dhs-16* may function in negative feedback by IIS, DA, or its metabolites.

These complex feedback circuits impact not only dauer formation and reproductive development but also lifespan at elevated temperatures. In *ttx-1* mutants, upregulation of hypodermal *daf-9* at 25°C fails and animals are short lived [54]. Short lifespan resembles that seen in *daf-9(rh50)* hypomorphs, and in both cases lifespan is restored to normal by loss of *daf-12*, suggesting that sufficient DA production is required to ensure lifespan-promoting activities of DAF-12 at elevated temperatures.

Although the DAs modulate nematode lifespan, whether small molecule bile acid-like metabolites can influence mammalian lifespan remains unknown. Elevated bile acid levels are associated with the long-lived Little mouse, as well as expression of xenobiotic detoxification genes, partially dependent upon FXRα [55]. Treatment of wild-type mice with cholic acid induces a similar xenobiotic detoxification expression profile. These studies suggest that BAs may act through FXRα to induce the xenobiotic response and impact lifespan, possibly as a means of longevity assurance. In worms as well, microarray analyses indicate that genes involved in xenobiotic metabolism are altered in long-lived *daf-2*/InsR and *daf-12(rh273)* ligand-binding domain mutants [56],[57]. Bile acids reduce serum triglycerides, increase insulin sensitivity, and decrease inflammation in animals fed high-fat diets [58]. Furthermore, polymorphisms in FXR may also be associated with reduced IGF levels and murine longevity [59]. In the future, it will be interesting to see if bile acid signaling plays a conserved role in impacting mammalian longevity.

## Materials and Methods

### *C. elegans* Strains

Worms were grown on NGM agar seeded with the *E. coli* bacteria OP50 at 20°C unless noted otherwise [60]. NGM contains cholesterol at a 5 µg/mL concentration, which is omitted in low cholesterol conditions. Strains were outcrossed at least three times prior to use. The following genotypes were used: N2, *daf-36(k114)*, *dhs-16(tm1890)*, *daf-9(k182)*, *daf-9(dh6)*, *daf-12(rh61rh411)*, *hsd-1(mg433)*, *ncr-1(nr2022)*, *daf-12(rh273)*, *daf-2(e1368)*, *daf-2(e1370)*, *daf-7(e1372)*, *daf-5(e1386)*, *daf-16(mu86)*, *dhs-16(tm1890) daf-12(rh61rh411)*, *dhs-16(tm1890) daf-16(mu86)*, *dhs-16(tm1890) daf-5(e1386)*, *dhs-16(tm1890) daf-9(k182)*, *dhs-16(tm1890) daf-36(k114)*, *dhs-16(tm1890) ncr-1(nr2022)*, *dhs-16(tm1890) hsd-1(mg433)*, *dhs-16(tm1890) daf-2(e1370)*, *daf-36(k114) daf-9(k182)*, *dhIs64(Pdaf-9::daf-9::gfp)*, *dhs-16(tm1890) dhIs64(Pdaf-9::daf-9::gfp)*, *daf-16(mu86) muIs109(Pdaf-16*::*gfp*::*daf-16;Podr-1::rfp)*, *dhs-16(tm1890) daf-16(mu86) muIs109(Pdaf-16*::*gfp*::*daf-16;Podr-1::rfp)*, *glp-1(e2141)*, *glp-1(e2141) daf-16(mu86) muIs109(Pdaf-16*::*gfp*::*daf-16;Podr-1::rfp)*, and *glp-1(e2141) dhs-16(tm1890) daf-16(mu86) muIs109(Pdaf-16*::*gfp*::*daf-16;Podr-1::rfp)*.

The extrachromosomal line *dhEx396*(*Pdhs-16::dhs-16::gfp;lin-15*[+]) was crossed into various mutant backgrounds for regulation analysis. Images of key tissues were taken at 40× magnification, calibrated with InSpeck Green Microscopy Image Intensity Calibration Beads (Molecular Probes), and comparison analysis was performed using ImageJ software (http://rsbweb.nih.gov/ij/). The *dhIs64* strain carries an integrated *Pdaf-9::daf-9::gfp*, previously described [40]. The same procedure was followed for these strains, and expression levels were categorized accordingly. *daf-16(mu86) muIs109(Pdaf-16::daf-16::gfp;Podr-1::rfp)* animals were kindly provided by Malene Hansen.

### Plasmids

For *dhs-16::gfp* construction, a 3.9 kb fragment containing the *dhs-16* coding region and 2.3 kb upstream promoter region was amplified with primers 5′-GCG GCCGCCTTCTCTCTTGCACCCTTGTTTGT-3′ (forward) and 5′-GGTACCTCAGAAACTGTAACATTATG-3′ (reverse) and cloned into pCRII-TOPO vector. *Kpn*I/*Not*I-digested *dhs-16*-TOPO was then inserted into the *Kpn*I/*Not*I-digested *gfp* vector L3871 (Fire vector kit 1997). The construct was injected with the *lin-15*[+] marker into *lin-15(n765)* mutant animals, and F1 animals were selected by rescue of the syn-muv phenotype. Two independent lines displayed similar expression patterns. The *dhEx396* extrachromosomal line fully rescued the gonadal migration defects and dauer-constitutive phenotypes of the *dhs-16(tm1890)* mutant animals.

### Genetic Screens

RNAi screens were carried out essentially as described [61]. RNAi clones were plated in 12-well plates, two *daf-36(k114)* hermaphrodites placed in each well to lay eggs, and grown at 25°C. Mig and Daf phenotypes were scored in L4/young adult animals of the next generation. Screens were carried out on the whole genome. Other genes identified in the screen will be described elsewhere.

### Isolation of Microsomes

Isolation of microsomes from HEK293T cells was performed as described [62]. Cells were grown in T-75 flasks and transfected with vector only or FLAG-tagged DHS-16. Expression of DHS-16 was verified by immunoblot, using anti-FLAG antibodies (Sigma). Microsomal fractions were resuspended in 0.1 M KPO_4_ buffer, pH 7.4, containing 1 mM EDTA and 20% glycerol, and stored at −80°C.

### Microsomal Incubations

Microsomes were thawed on ice and brought to 80 µg/mL in 0.1 M KPO_4_ buffer, pH 7.4, containing 1 mM EDTA. Substrates were added at 100 µM in 0.5 mL total volume, pre-incubated for 3 min at 37°C, and reacted with 1 mM NAD for 16 h at 37°C. Reactions were processed by extracting twice with 2 mL MTBE, combining the top layers and drying under nitrogen. 0.5 µg of cholesterol-*d_7_* was added as an internal standard for extractions.

### Rescue Assays

Microsomal extracts from three incubations were combined and resuspended in 50 µL methanol, mixed with 5× concentrated OP50, vacuum dried, resuspended in 100 µL 1× OP50, and plated on 3 cm plates containing 3 mL NGM agar. For rescue, ∼100 embryos from a 4–8 h egg laying were transferred onto the bacterial lawn, and scored for dauer arrest at 27°C after 48 h. For rescue experiments with pure steroids, 10 µL compounds in ethanol (or ethanol alone) were mixed with 40 µL 5× concentrated OP50 bacteria and plated. Final concentrations include the total volume of agar (3 mL). Dauer arrest was scored after 60 h at 20°C and after 48 h at 25°C and 27°C.

### Nematode Lipid Extracts

Worms were grown on twenty 10 cm NGM agar plates seeded with OP50 bacteria. Gravid adults were bleached and the resulting embryos transferred to liquid culture (S-complete medium supplemented with 100× concentrated OP50). Two to three successive rounds of growth and lysis were performed. For the final round, worms were grown at 20°C until the L3-L4 stage, harvested, frozen in liquid nitrogen, and stored at −80°C. Thawed worms were homogenized by sonication, and total lipids (plus 1 µg cholesterol-*d_7_* or CDCA-*d_4_*/10^7^ worms) were extracted with 2∶1 chloroform∶methanol. The resulting chloroform layer was dried under nitrogen. For GC/MS/MS analysis, growth and lysis of worms was carried out on 10 cm NGM plates.

### LC/MS/MS Analysis

Samples were analyzed by LC/MS/MS using 6410 Triple Quadrupole LC/MS instrument (Agilent Technologies) equipped with an ESI source in positive ion mode. Samples were dissolved in methanol, spiked with cholesterol-*d_7_* as an internal standard, and separated on a Zorbax XDB-C18 column (4.6×50 mm, 3.5 µm) at 0.4 mL/min. The mobile phase consisted of HPLC grade water (A) and methanol (B) both containing 5 mM NH_4_Ac. The following gradient was run: 0–1 min, 90% (B); 1–3.3 min, 90% to 100% (B); 3.3–20 min, 100% (B). MS parameters were as follows: gas temperature 175°C, nebulizer pressure 35 psg, drying gas (nitrogen) 10 L/min, VCap 4,000 V (positive) and 6,000 V (negative), and column temperature 40°C. Using MRM monitoring (in positive-ion mode) the following transitions were observed: cholesterol-*d_7_* (*m/z* 411→376, RT 12.8 min), lathosterone (*m/z* 402→385, RT 13.9 min), lathosterol (*m/z* 404→369, RT 12.6 min), 7-dehydrocholesterol (*m/z* 385→369, RT 11.7 min), and 4-cholesten-3-one (*m/z* 385→109, RT 12.7 min). Fragmentor voltage and collision energy settings for each compound are summarized in Table S3.

### GC/MS/MS Analysis

Dafachronic acid levels were analyzed by GC/MS/MS on a 7000A Triple Quadrupole GC/MS instrument (Agilent Technologies) equipped with an ESI source and an HP-5ms column. Briefly, lipid extracts were spiked with 5β-cholanic acid as an internal standard, derivitized with trimethylsilyldiazomethane, and analyzed in MRM mode. The following transitions were observed: 5β-cholanic acid (*m/z* 374.3→264.0) and Δ^7^-dafachronic acid (*m/z* 428.3→229.1). The methods used for analysis of all other compounds will be described elsewhere.

### Lifespan Analysis

Adult lifespan assays and gonadal cell ablations were performed as previously described [25]. Day 0 corresponds to the L4 stage. Exploded and egg-laying defective animals were excluded from the analysis. Statistical analyses were performed using the log-rank (Mantel-Cox) method with GraphPad Prism software.

### DAF-16::GFP Nuclear Localization Experiments

Strains carrying the integrated *muIs109(daf-16P::daf-16::gfp)* array were used in germline ablations and placed at 20°C. After 4 d, at day 1 of adulthood, worms were scored for nuclear localization of DAF-16::GFP in intestinal cells under a dissection microscope. Worms with a clearly dotted appearance throughout the body were scored as strongly localized. In *glp-1* experiments, worms were kept at the restrictive temperature of 25°C from eggs until scoring at day 1 of adulthood, and two of three experiments were performed blind. Images of the anterior intestinal cells were taken at 40× magnification and the relative expression intensity of individual nuclei versus equal areas of adjacent cytoplasm was determined using Image J software (http://rsbweb.nih.gov/ij/).

### qRT-PCR

Real-time quantification for microRNAs by RT-PCR was performed with a protocol modified from a previous report [63]. Briefly, total RNA was purified from L3 stage larvae using TRIzol (Invitrogen) and the miRNeasy kit (Qiagen). TaqMan MicroRNA Reverse Transcription kit (Applied Biosystems) was used to generate cDNA with microRNA-specific primers. qRT-PCR was performed with Power SYBR Green master mix (Applied Biosystems) according to the manufacturer's instructions. Sno-RNA U18 was used as an internal control. The following primers were used: 5′-CAGTGCAGGGTCCGAGGT-3′ (*U18-RT*); 5′-GGCAGTGATGATCACAAATC-3′(*U18*-f); 5′-TGGCTCAGCCGGTTTTCTAT-3′ (*U18*-r); 5′-GTCGTATCCAGTGCAGGGTCCGAGGTATTCGCACTGGATACGACTCATTT-3′ (*mir-241*-RT); 5′-CGCTGAGGTAGGTGCGAG-3′ (*mir-241*-f); and 5′-GTGCAGGGTCCGAGGT-3′ (microRNA reverse primer).

### Phylogenetic Tree and Multiple Sequence Alignment

Sequences were retrieved using PSI-BLAST [64] with filter turned on. Reciprocal BLASTs were used to determine orthologous relationships between human, nematode, and fly proteins. HomoloGene [65] was used wherever available. Multiple sequence alignments were done with ClustalX [66] using standard parameters. For tree building, the multiple sequence alignment was trimmed using the GBlocks server [67] with relaxed settings. The tree was calculated using Phyml [68] with standard settings and 500 bootstrap steps. The resulting tree was displayed in Dendroscope [69] and prepared for publication using Adobe Illustrator. The sequence of *A. suum* DHS-16 was determined by BLAST against the expressed-sequence tag database for *A. suum* and sequencing cDNA fragments amplified from *A. suum* adult head cDNA (provided by the laboratory of Angela Mousley, Queen's University Belfast, Northern Ireland, U.K.). The sequence was submitted to NCBI and has received the following GenBank accession number: JF753272.

### Statistical Analysis

Results are presented as M ± SD or SEM, as indicated. *p* values were calculated using GraphPad Prism software by Student's *t* test.

## Supporting Information

Figure S1 **Phylogenetic tree and multiple sequence alignment of DHS-16 and homologs.** (A) Phylogenetic tree displaying the evolutionary relationships between *C. elegans* DHS-16 and related SDR enzymes. Clades are formed according to phylum, preventing unambiguous interpretation of orthology relationships. *A. suum* DHS-16 is clearly the ortholog of the DHS-16 proteins of other nematodes. Arthropod relatives include Shroud and CG8888, although these show substantial divergence. There was also a notable expansion of Retinol Dehydrogenase 16-type enzymes in mouse. Species abbreviations are as follows: *As*, *Ascaris suum*; *Ce*, *Caenorhabditis elegans*; *Cr*, *Caenorhabditis remanei*; *Dm*, *Drosophila melanogaster*; *Hs*, *Homo sapiens*; *Mm*, *Mus musculus*; *Tc*, *Tribolium castaneum*. Accession numbers are as follows: *As* DHS-16: JF753272; *Ce* DHS-16: NP_504554; *Ce* DHS-20: NP_505941; *Ce* DHS-2: NP_491575; *Cr* DHS-16: XP_003112544; *Cr* DHS-20: XP_003113962; *Cr* DHS-2: XP_003112163; *Dm* Shroud: NP_651725; *Dm* CG8888: NP_610724; *Tc* Shroud: XP_973118; *Tc* CG8888: XP_967401; *Mm* SDR9C7: NP_081577; *Mm* HSD17B6: NP_038814; *Mm* RD1: NP_536684; *Mm* RD5: NP_598767; *Mm* RD16: NP_033066; *Mm* RD9: NP_694773; *Mm* RD9: NP_694773; *Mm* RD2: NP_671755; *Mm* R3aHSD: NP_663399; *Mm* SDR9: NP_780721; *Hs* SDR9C7: NP_683695; *Hs* HSD17B6: NP_003716; *Hs* RD16: NP_003699; *Hs* SDR9: NP_005762; *Hs* 11cRD: NP_002896. (B) Multiple Sequence Alignment of the DHS-16 protein with putative orthologs in humans and *Ascaris suum* as well as with the closely related *C. elegans* DHS-2 and DHS-20. Identical residues are highlighted in bright yellow and those that are conserved in dark yellow. The SDR/NAD(P)-Binding Rossmann domain is highlighted in light blue and according to its position in *C. elegans* DHS-16.
(TIF)

Figure S2 **Gene structure of the** ***dhs-16*** **locus.** (A) The genomic environs of the *dhs-16* locus on Chromosome V are displayed. The cosmid C10F3 (blue) contains the *dhs-16* sequence (C10F3.2). Below, the structure of the *dhs-16* gene is shown, which consists of 3 exons and 2 introns, the SDR/NAD(P)-Binding Rossman fold domain (green) and two predicted transmembrane domains (blue). The *tm1890* allele (red) is a 607 bp deletion spanning the first exon and is a predicted null allele; the flanking sequences are shown. (B) The structure of the C-terminal *dhs-16::gfp* fusion construct used in expression analyses.
(TIF)

Figure S3 **Additional rescue experiments provide predictions of DA synthesis.** (A) Proposed precursors of the DAs (33 µM) do not rescue the Daf-c phenotypes of the *daf-12(rh273)* ligand-binding domain mutant (*N* = 2, M ± SD). (B) N2 wild-type animals do not display Daf-c phenotypes at 27°C on the empty vehicle ethanol or any of the compounds tested (*N*≥3, M ± SD). (C) Lathosterone and the DAs give more efficient rescue of *dhs-16(tm1890)* mutants (***p<0.0001) compared to 4-cholesten-3-one (**p<0.01) at nanomolar concentrations (250 nM) (*N*≥3, M ± SD). (D) *daf-36(k114)* mutant animals are rescued with 7-dehydrocholesterol and proposed downstream precursors of the DAs (*N* = 3, M ± SD; ***p<0.0001). (E) Rescue of *dhs-16;daf-36* double mutants is similar to *dhs-16* single mutant animals, consistent with a role of *dhs-16* downstream of *daf-36* (*N* = 3, M ± SD; ***p<0.0001). (F) Rescue of *dhs-16;hsd-1(mg433)* double mutant dauer formation is also similar to *dhs-16* single mutant animals (*N* = 3, M ± SD; ***p<0.0001). *hsd-1* single mutants do not form dauers under these conditions.
(TIF)

Figure S4 **Influence of** ***hsd-1*** **on DA metabolites.** (A) Complete GC/MS/MS analysis of sterol levels in L3-stage N2 wild-type, *dhs-16(tm1890)*, *hsd-1(mg433)*, and *dhs-16;hsd-1* double mutants at 20°C reveals that no significant changes in the proposed DA precursors are present in *hsd-1* animals, and that it is not required for 4-cholesten-3-one production as previously proposed. HSD-1 likely acts in a parallel pathway, possibly making another as yet unknown ligand for DAF-12. Double mutants display deficiencies in lathosterone and 4-methyl sterols at 20°C, but not Δ^7^-dafachronic acid (*N*≥6, M ± SEM; **below detection limit, *p<0.05). (B) Levels of lathosterone, 4-cholesten-3-one, and Δ^7^-dafachronic acid at the elevated temperature of 25°C relative to N2 wild-type. No significant change in lathosterone or 4-cholesten-3-one is noted in *hsd-1* single mutants, whereas DA levels are elevated relative to wild-type. *dhs-16;hsd-1* double mutants display decreased levels of DA, corresponding with the more severe L2d and dauer phenotypes displayed by these animals, but show increased levels of 4-cholesten-3-one, presumably due to feedback (*N*≥3, M ± SEM; **below detection limit, *p<0.05).
(TIF)

Figure S5 **Homeostatic feedback on hypodermal** ***daf-9*** **expression.** (A) *daf-9::gfp* hypodermal expression is upregulated in *dhs-16(tm1890)* mutants at 20°C (***p<0.0001). Lathosterone and the DAs fully rescue this upregulation, while proposed upstream precursors do not (**p<0.01). The fraction of animals with strong (green), weak (yellow), or no (red) hypodermal GFP expression is shown (*N*≥3, M ± SD). (B) At 27°C, *dhs-16* mutant animals enter dauer diapause, in which *daf-9::gfp* hypodermal expression is downregulated. Animals that do not enter dauer display higher levels of *daf-9* expression than N2 wild-type, as seen at 20°C (**p<0.01). Feeding lathosterone rescues the dauer phenotype and restores *daf-9::gfp* expression to wild-type levels (*N*≥3, M ± SD; **p<0.01).
(TIF)

Figure S6 **RNAi knockdown of** ***dhs-16*** **reduces DAF-16::GFP localization in germline-less mutant animals.** Treatment of *glp-1(e2141ts)* animals with *dhs-16* RNAi leads to a reduction in strongly nuclear-localized intestinal DAF-16::GFP in day 1 adults at the restrictive temperature of 25°C. Percent animals with strong localization (green) and weak localization (yellow) are displayed. Localization is restored upon provision of lathosterone or Δ^7^-dafachronic acid, but not with lathosterol (*N* = 3, M ± SD; **p<0.01, *p<0.05).
(TIF)

Table S1 **Knockdown of** ***emb-8*** **leads to DA deficiency-associated phenotypes.**
(DOC)

Table S2 **Longevity in the absence of the germline is partially dependent upon** ***dhs-16***.
(DOC)

Table S3 **Settings for LC/MS/MS analysis of DA precursors.**
(DOC)

## Abbreviations

DA: dafachronic acid
Daf-c: Dauer-constitutive
Daf-d: Dauer-defective
NHR: nuclear hormone receptor

## References

[1] Kenyon C. J (2010). The genetics of ageing.. Nature.

[2] Eisenberg T, Knauer H, Schauer A, Buttner S, Ruckenstuhl C (2009). Induction of autophagy by spermidine promotes longevity.. Nat Cell Biol.

[3] Honda Y, Tanaka M, Honda S (2010). Trehalose extends longevity in the nematode *Caenorhabditis elegans*.. Aging Cell.

[4] Lucanic M, Held J. M, Vantipalli M. C, Klang I. M, Graham J. B (2011). N-acylethanolamine signalling mediates the effect of diet on lifespan in *Caenorhabditis elegans*.. Nature.

[5] Goudeau J, Bellemin S, Toselli-Mollereau E, Shamalnasab M, Chen Y (2011). Fatty Acid Desaturation Links Germ Cell Loss to Longevity Through NHR-80/HNF4 in *C. elegans*.. PLoS Biol.

[6] Mangelsdorf D. J, Thummel C, Beato M, Herrlich P, Schutz G (1995). The Nuclear Receptor Superfamily: The Second Decade.. Cell.

[7] Wollam J, Antebi A (2011). Sterol Regulation of Metabolism, Homeostasis, and Development.. Annu Rev Biochem.

[8] Lefebvre P, Cariou B, Lien F, Kuipers F, Staels B (2009). Role of Bile Acids and Bile Acid Receptors in Metabolic Regulation.. Physiol Rev.

[9] Kalaany N. Y, Mangelsdorf D. J (2006). LXRs and FXR: The Yin and Yang of Cholesterol and Fat Metabolism.. Annu Rev Physiol.

[10] Houten S. M, Watanabe M, Auwerx J (2006). Endocrine functions of bile acids.. EMBO J.

[11] Motola D. L, Cummins C. L, Rottiers V, Sharma K. K, Li T (2006). Identification of Ligands for DAF-12 that Govern Dauer Formation and Reproduction in *C. elegans*.. Cell.

[12] Fielenbach N, Antebi A (2008). *C. elegans* dauer formation and the molecular basis of plasticity.. Genes Dev.

[13] Antebi A, Yeh W. H, Tait D, Hedgecock E. M, Riddle D. L (2000). *daf-12* encodes a nuclear receptor that regulates the dauer diapause and developmental age in *C. elegans*.. Genes Dev.

[14] Riddle D. L, Swanson M. M, Albert P. S (1981). Interacting genes in nematode dauer larva formation.. Nature.

[15] Hsin H, Kenyon C (1999). Signals from the reproductive system regulate the lifespan of *C. elegans*.. Nature.

[16] Kenyon C (2010). A pathway that links reproductive status to lifespan in *Caenorhabditis elegans*.. Ann N Y Acad Sci.

[17] Gerisch B, Rottiers V, Li D, Motola D. L, Cummins C. L (2007). A bile acid-like steroid modulates *Caenorhabditis elegans* lifespan through nuclear receptor signaling.. Proc Natl Acad Sci U S A.

[18] Held J. M, White M. P, Fisher A. L, Gibson B. W, Lithgow G. J (2006). DAF-12-dependent rescue of dauer formation in *Caenorhabditis elegans* by (25S)-cholestenoic acid.. Aging Cell.

[19] Gems D, Sutton A. J, Sundermeyer M. L, Albert P. S, King K. V (1998). Two Pleiotropic Classes of *daf-2* Mutation Affect Larval Arrest, Adult Behavior, Reproduction and Longevity in *Caenorhabditis elegans*.. Genetics.

[20] Larsen P. L, Albert P. S, Riddle D. L (1995). Genes that Regulate Both Development and Longevity in *Caenorhabditis elegans*.. Genetics.

[21] Ludewig A. H, Kober-Eisermann C, Weitzel C, Bethke A, Neubert K (2004). A novel nuclear receptor/coregulator complex controls *C. elegans* lipid metabolism, larval development, and aging.. Genes Dev.

[22] Wang Z, Zhou X. E, Motola D. L, Gao X, Suino-Powell K (2009). Inaugural Article: Identification of the nuclear receptor DAF-12 as a therapeutic target in parasitic nematodes.. Proc Natl Acad Sci U S A.

[23] Ogawa A, Streit A, Antebi A, Sommer R. J (2009). A Conserved Endocrine Mechanism Controls the Formation of Dauer and Infective Larvae in Nematodes.. Curr Biol.

[24] Rottiers V, Motola D. L, Gerisch B, Cummins C. L, Nishiwaki K (2006). Hormonal Control of *C. elegans* Dauer Formation and Life Span by a Rieske-like Oxygenase.. Dev Cell.

[25] Gerisch B, Weitzel C, Kober-Eisermann C, Rottiers V, Antebi A (2001). A Hormonal Signaling Pathway Influencing *C. elegans* Metabolism, Reproductive Development, and Life Span.. Dev Cell.

[26] Jia K, Albert P. S, Riddle D. L (2002). DAF-9, a cytochrome P450 regulating *C. elegans* larval development and adult longevity.. Development.

[27] Patel D. S, Fang L. L, Svy D. K, Ruvkun G, Li W (2008). Genetic identification of HSD-1, a conserved steroidogenic enzyme that directs larval development in *Caenorhabditis elegans*.. Development.

[28] Dumas K. J, Guo C, Wang X, Burkhart K. B, Adams E. J (2010). Functional divergence of dafachronic acid pathways in the control of *C. elegans* development and lifespan.. Dev Biol.

[29] Wollam J, Magomedova L, Magner D. B, Shen Y, Rottiers V (2011). The Rieske oxygenase DAF-36 functions as a cholesterol 7-desaturase in steroidogenic pathways governing longevity.. Aging Cell.

[30] Li J, Brown G, Ailion M, Lee S, Thomas J. H (2004). NCR-1 and NCR-2, the *C. elegans* homologs of the human Niemann-Pick type C1 disease protein, function upstream of DAF-9 in the dauer formation pathways.. Development.

[31] Kavanagh K. L, Jornvall H, Persson B, Oppermann U (2008). Medium- and short-chain dehydrogenase/reductase gene and protein families : the SDR superfamily: functional and structural diversity within a family of metabolic and regulatory enzymes.. Cell Mol Life Sci.

[32] Kowalik D, Haller F, Adamski J, Moeller G (2009). In search for function of two human orphan SDR enzymes: Hydroxysteroid dehydrogenase like 2 (HSDL2) and short-chain dehydrogenase/reductase-orphan (SDR-O).. J Steroid Biochem Mol Biol.

[33] Chen W, Song M. S, Napoli J. L (2002). *SDR-O*: an orphan short-chain dehydrogenase/reductase localized at mouse chromosome 10/human chromosome 12.. Gene.

[34] Omura T (2010). Structural diversity of cytochrome P450 enzyme system.. J Biochem.

[35] Rappleye C. A, Tagawa A, Le Bot N, Ahringer J, Aroian R. V (2003). Involvement of fatty acid pathways and cortical interaction of the pronuclear complex in *Caenorhabditis elegans* embryonic polarity.. BMC Dev Biol.

[36] Kulas J, Schmidt C, Rothe M, Schunck W. H, Menzel R (2008). Cytochrome P450-dependent metabolism of eicosapentaenoic acid in the nematode *Caenorhabditis elegans*.. Arch Biochem Biophys.

[37] Leung M. C, Goldstone J. V, Boyd W. A, Freedman J. H, Meyer J. N (2010). *Caenorhabditis elegans* Generates Biologically Relevant Levels of Genotoxic Metabolites from Aflatoxin B1 but Not Benzo[a]pyrene *in vivo*.. Toxicol Sci.

[38] Sharma K. K, Wang Z, Motola D. L, Cummins C. L, Mangelsdorf D. J (2009). Synthesis and Activity of Dafachronic Acid Ligands for the *C. elegans* DAF-12 Nuclear Hormone Receptor.. Mol Endocrinol.

[39] Hannich J. T, Entchev E. V, Mende F, Boytchev H, Martin R (2009). Methylation of the Sterol Nucleus by STRM-1 Regulates Dauer Larva Formation in *Caenorhabditis elegans*.. Dev Cell.

[40] Gerisch B, Antebi A (2004). Hormonal signals produced by DAF-9/cytochrome P450 regulate *C. elegans* dauer diapause in response to environmental cues.. Development.

[41] Mak H. Y, Ruvkun G (2004). Intercellular signaling of reproductive development by the *C. elegans* DAF-9 cytochrome P450.. Development.

[42] Matyash V, Geier C, Henske A, Mukherjee S, Hirsh D (2001). Distribution and Transport of Cholesterol in *Caenorhabditis elegans*.. Mol Biol Cell.

[43] Arantes-Oliveira N, Apfeld J, Dillin A, Kenyon C (2002). Regulation of Life-Span by Germ-Line Stem Cells in *Caenorhabditis elegans*.. Science.

[44] Rottiers V, Antebi A (2006). Control of *Caenorhabditis elegans* life history by nuclear receptor signal transduction.. Exp Gerontol.

[45] Yamawaki T. M, Berman J. R, Suchanek-Kavipurapu M, McCormick M, Maria Gaglia M (2010). The Somatic Reproductive Tissues of *C. elegans* Promote Longevity through Steroid Hormone Signaling.. PLoS Biol.

[46] Lin K, Hsin H, Libina N, Kenyon C (2001). Regulation of the *Caenorhabditis elegans* longevity protein DAF-16 by insulin/IGF-1 and germline signaling.. Nat Genet.

[47] Berman J. R, Kenyon C (2006). Germ-Cell Loss Extends *C. elegans* Life Span through Regulation of DAF-16 by *kri-1* and Lipophilic-Hormone Signaling.. Cell.

[48] Yoshiyama T, Namiki T, Mita K, Kataoka H, Niwa R (2006). Neverland is an evolutionally conserved Rieske-domain protein that is essential for ecdysone synthesis and insect growth.. Development.

[49] Yoshiyama-Yanagawa T, Enya S, Shimada-Niwa Y, Yaguchi S, Haramoto Y (2011). The conserved Rieske oxygenase DAF-36/Neverland is a novel cholesterol metabolizing enzyme.. J Biol Chem.

[50] Russell D. W (2003). The enzymes, regulation, and genetics of bile acid synthesis.. Annu Rev Biochem.

[51] Butcher R. A, Ragains J. R, Li W, Ruvkun G, Clardy J (2009). Biosynthesis of the *Caenorhabditis elegans* dauer pheromone.. Proc Natl Acad Sci U S A.

[52] Yabe T, Suzuki N, Furukawa T, Ishihara T, Katsura I (2005). Multidrug resistance-associated protein MRP-1 regulates dauer diapause by its export activity in *Caenorhabditis elegans*.. Development.

[53] Sinal C. J, Tohkin M, Miyata M, Ward J. M, Lambert G (2000). Targeted Disruption of the Nuclear Receptor FXR/BAR Impairs Bile Acid and Lipid Homeostasis.. Cell.

[54] Lee S. J, Kenyon C (2009). Regulation of the Longevity Response to Temperature by Thermosensory Neurons in *Caenorhabditis elegans*.. Curr Biol.

[55] Amador-Noguez D, Dean A, Huang W, Setchell K, Moore D (2007). Alterations in xenobiotic metabolism in the long-lived Little mice.. Aging Cell.

[56] Fisher A. L, Lithgow G. J (2006). The nuclear hormone receptor DAF-12 has opposing effects on *Caenorhabditis elegans* lifespan and regulates genes repressed in multiple long-lived worms.. Aging Cell.

[57] McElwee J. J, Schuster E, Blanc E, Thomas J. H, Gems D (2004). Shared Transcriptional Signature in *Caenorhabditis elegans* Dauer Larvae and Long-lived *daf-2* Mutants Implicates Detoxification System in Longevity Assurance.. J Biol Chem.

[58] Hylemon P. B, Zhou H, Pandak W. M, Ren S, Gil G (2009). Bile acids as regulatory molecules.. J Lipid Res.

[59] Leduc M. S, Hageman R. S, Meng Q, Verdugo R. A, Tsaih S. W (2010). Identification of genetic determinants of IGF-1 levels and longevity among mouse inbred strains.. Aging Cell.

[60] Brenner S (1974). The Genetics of *Caenorhabditis elegans*.. Genetics.

[61] Fraser A. G, Kamath R. S, Zipperlen P, Martinez-Campos M, Sohrmann M (2000). Functional genomic analysis of *C. elegans* chromosome I by systematic RNA interference.. Nature.

[62] Bozidis P, Williamson C. D, Colberg-Poley A. M (2007). Isolation of Endoplasmic Reticulum, Mitochondria, and Mitochondria-Associated Membrane Fractions from Transfected Cells and from Human Cytomegalovirus-Infected Primary Fibroblasts.. Curr Protoc Cell Biol Chapter.

[63] Chen C, Ridzon D. A, Broomer A. J, Zhou Z, Lee D. H (2005). Real-time quantification of microRNAs by stem-loop RT-PCR.. Nucleic Acids Res.

[64] Altschul S. F, Madden T. L, Schaffer A. A, Zhang J, Zhang Z (1997). Gapped BLAST and PSI-BLAST: a new generation of protein database search programs.. Nucleic Acids Res.

[65] Sayers E. W, Barrett T, Benson D. A, Bolton E, Bryant S. H (2011). Database resources of the National Center for Biotechnology Information.. Nucleic Acids Res.

[66] Chenna R, Sugawara H, Koike T, Lopez R, Gibson T. J (2003). Multiple sequence alignment with the Clustal series of programs.. Nucleic Acids Res.

[67] Talavera G, Castresana J (2007). Improvement of Phylogenies after Removing Divergent and Ambiguously Aligned Blocks from Protein Sequence Alignments.. Syst Biol.

[68] Guindon S, Delsuc F, Dufayard J. F, Gascuel O (2009). Estimating Maximum Likelihood Phylogenies with PhyML.. Methods Mol Biol.

[69] Huson D. H, Richter D. C, Rausch C, Dezulian T, Franz M (2007). Dendroscope: An interactive viewer for large phylogenetic trees.. BMC Bioinformatics.

