# Supplementary figures and images for: A Novel 3-Hydroxysteroid Dehydrogenase That Regulates Reproductive Development and Longevity
Source: PLoS Biol. 2012 Apr 10;10(4):e1001305. doi: 10.1371/journal.pbio.1001305 (PMC3323522; doi:10.1371/journal.pbio.1001305)

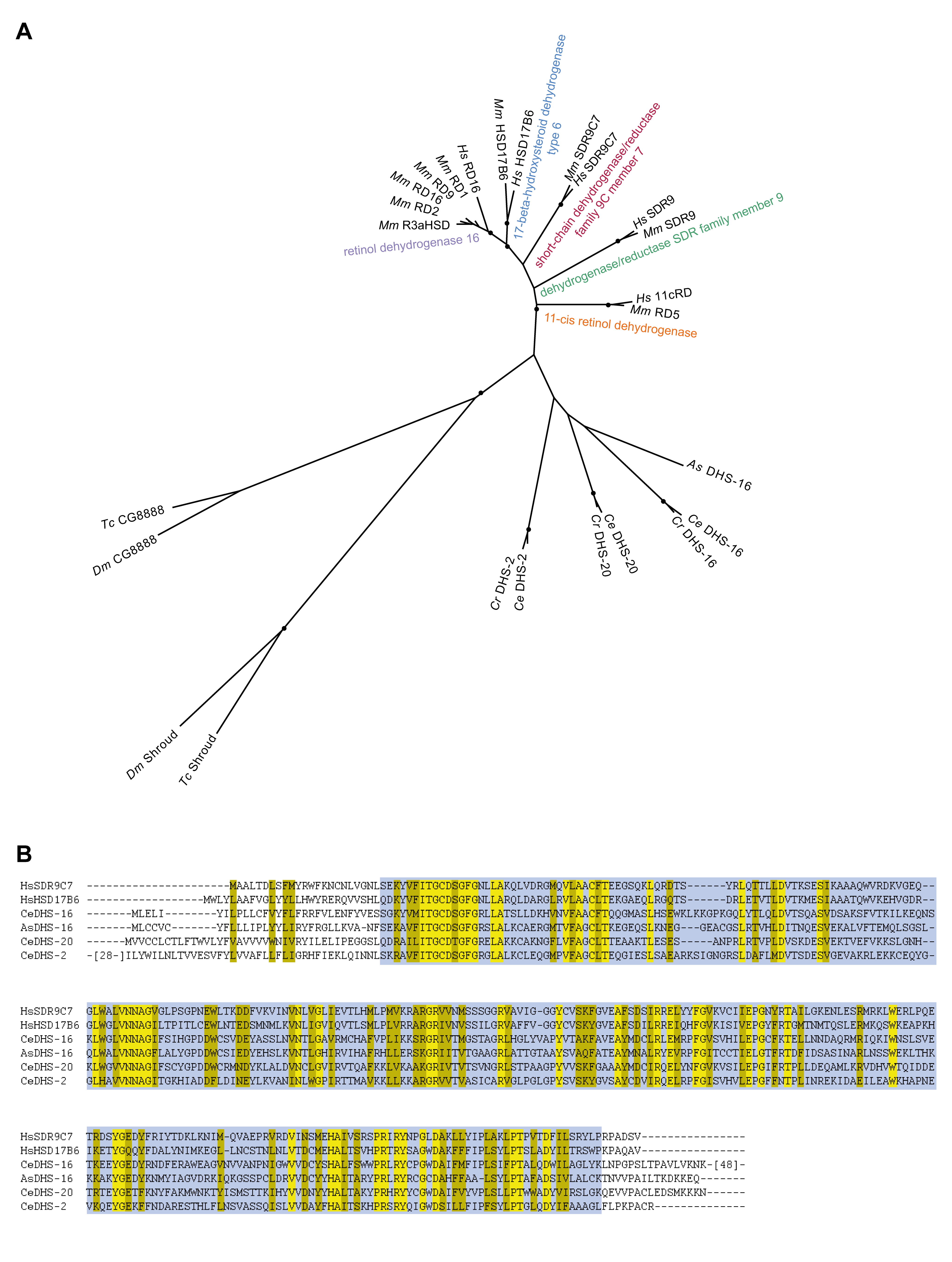

Supplement: Figure S1 — Phylogenetic tree and multiple sequence alignment of DHS-16 and homologs. (A) Phylogenetic tree displaying the evolutionary relationships between C. elegans DHS-16 and related SDR enzymes. Clades are formed according to phylum, preventing unambiguous interpretation of orthology relationships. A. suum DHS-16 is clearly the ortholog of the DHS-16 proteins of other nematodes. Arthropod relatives include Shroud and CG8888, although these show substantial divergence. There was also a notable expansion of Retinol Dehydrogenase 16-type enzymes in mouse. Species abbreviations are as follows: As, Ascaris suum; Ce, Caenorhabditis elegans; Cr, Caenorhabditis remanei; Dm, Drosophila melanogaster; Hs, Homo sapiens; Mm, Mus musculus; Tc, Tribolium castaneum. Accession numbers are as follows: As DHS-16: JF753272; Ce DHS-16: NP_504554; Ce DHS-20: NP_505941; Ce DHS-2: NP_491575; Cr DHS-16: XP_003112544; Cr DHS-20: XP_003113962; Cr DHS-2: XP_003112163; Dm Shroud: NP_651725; Dm CG8888: NP_610724; Tc Shroud: XP_973118; Tc CG8888: XP_967401; Mm SDR9C7: NP_081577; Mm HSD17B6: NP_038814; Mm RD1: NP_536684; Mm RD5: NP_598767; Mm RD16: NP_033066; Mm RD9: NP_694773; Mm RD9: NP_694773; Mm RD2: NP_671755; Mm R3aHSD: NP_663399; Mm SDR9: NP_780721; Hs SDR9C7: NP_683695; Hs HSD17B6: NP_003716; Hs RD16: NP_003699; Hs SDR9: NP_005762; Hs 11cRD: NP_002896. (B) Multiple Sequence Alignment of the DHS-16 protein with putative orthologs in humans and Ascaris suum as well as with the closely related C. elegans DHS-2 and DHS-20. Identical residues are highlighted in bright yellow and those that are conserved in dark yellow. The SDR/NAD(P)-Binding Rossmann domain is highlighted in light blue and according to its position in C. elegans DHS-16. (TIF) [file pbio.1001305.s001.tif]

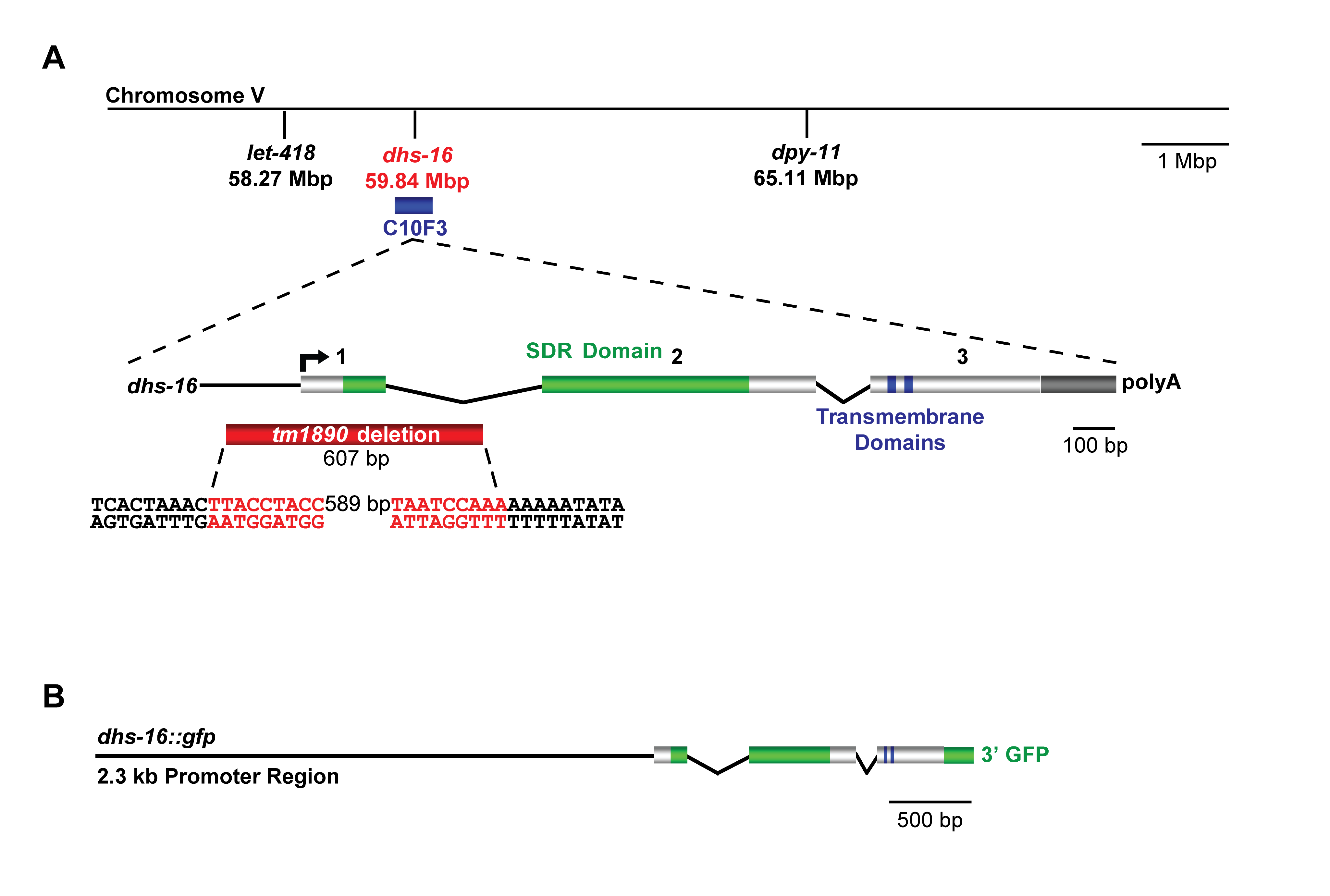

Supplement: Figure S2 — Gene structure of the dhs-16 locus. (A) The genomic environs of the dhs-16 locus on Chromosome V are displayed. The cosmid C10F3 (blue) contains the dhs-16 sequence (C10F3.2). Below, the structure of the dhs-16 gene is shown, which consists of 3 exons and 2 introns, the SDR/NAD(P)-Binding Rossman fold domain (green) and two predicted transmembrane domains (blue). The tm1890 allele (red) is a 607 bp deletion spanning the first exon and is a predicted null allele; the flanking sequences are shown. (B) The structure of the C-terminal dhs-16::gfp fusion construct used in expression analyses. (TIF) [file pbio.1001305.s002.tif]

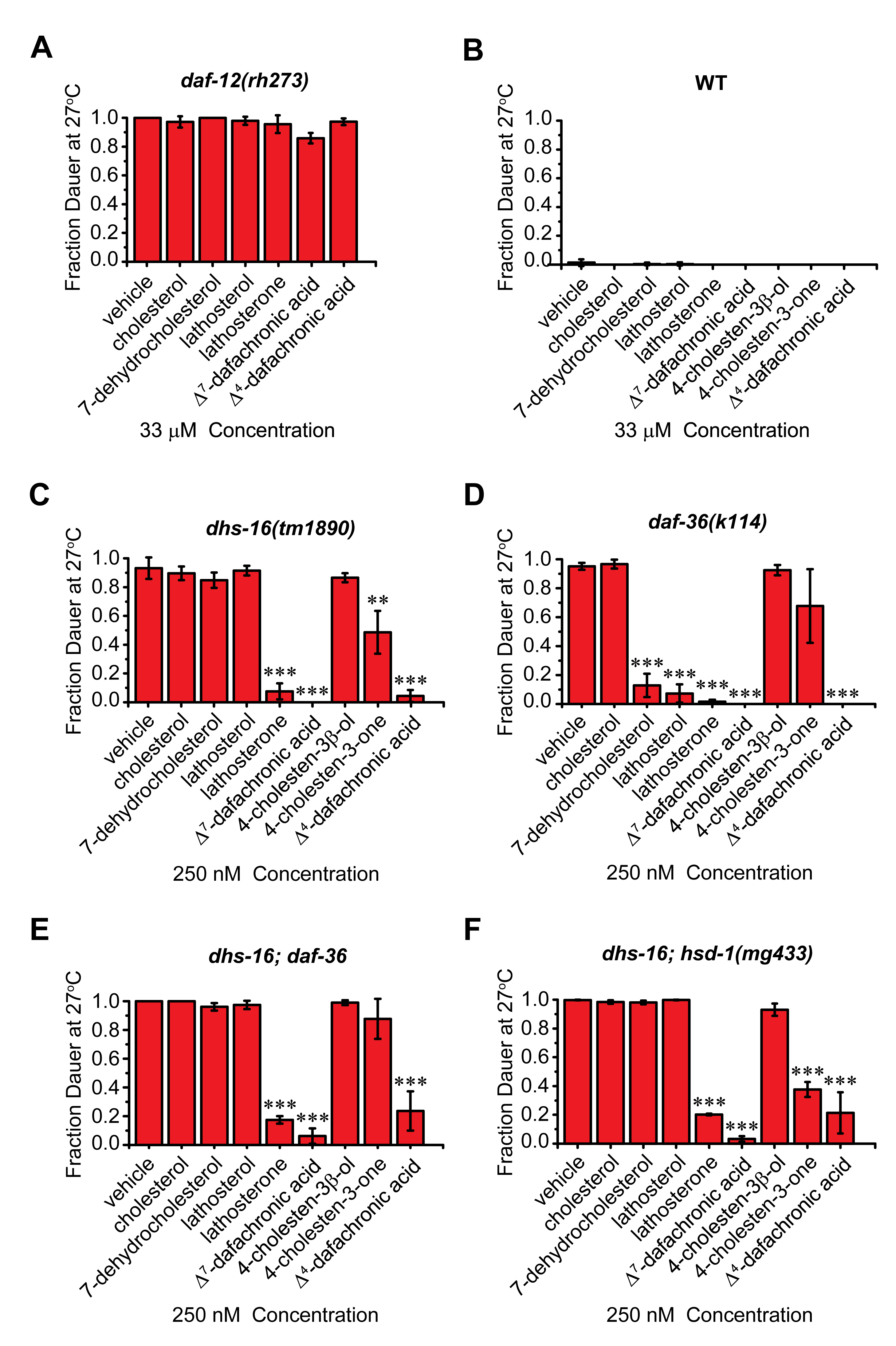

Supplement: Figure S3 — Additional rescue experiments provide predictions of DA synthesis. (A) Proposed precursors of the DAs (33 µM) do not rescue the Daf-c phenotypes of the daf-12(rh273) ligand-binding domain mutant (N = 2, M ± SD). (B) N2 wild-type animals do not display Daf-c phenotypes at 27°C on the empty vehicle ethanol or any of the compounds tested (N≥3, M ± SD). (C) Lathosterone and the DAs give more efficient rescue of dhs-16(tm1890) mutants (***p<0.0001) compared to 4-cholesten-3-one (**p<0.01) at nanomolar concentrations (250 nM) (N≥3, M ± SD). (D) daf-36(k114) mutant animals are rescued with 7-dehydrocholesterol and proposed downstream precursors of the DAs (N = 3, M ± SD; ***p<0.0001). (E) Rescue of dhs-16;daf-36 double mutants is similar to dhs-16 single mutant animals, consistent with a role of dhs-16 downstream of daf-36 (N = 3, M ± SD; ***p<0.0001). (F) Rescue of dhs-16;hsd-1(mg433) double mutant dauer formation is also similar to dhs-16 single mutant animals (N = 3, M ± SD; ***p<0.0001). hsd-1 single mutants do not form dauers under these conditions. (TIF) [file pbio.1001305.s003.tif]

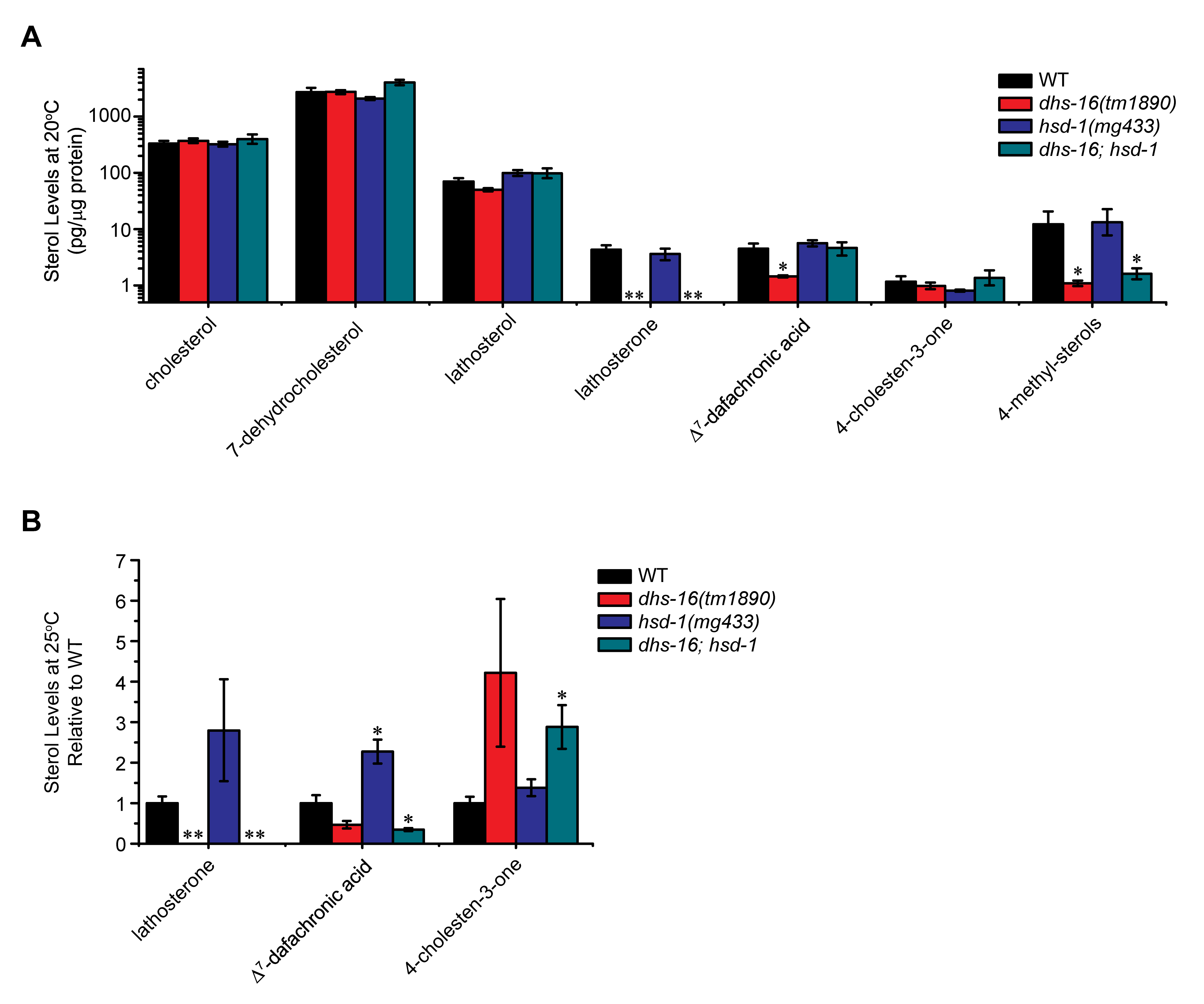

Supplement: Figure S4 — Influence of hsd-1 on DA metabolites. (A) Complete GC/MS/MS analysis of sterol levels in L3-stage N2 wild-type, dhs-16(tm1890), hsd-1(mg433), and dhs-16;hsd-1 double mutants at 20°C reveals that no significant changes in the proposed DA precursors are present in hsd-1 animals, and that it is not required for 4-cholesten-3-one production as previously proposed. HSD-1 likely acts in a parallel pathway, possibly making another as yet unknown ligand for DAF-12. Double mutants display deficiencies in lathosterone and 4-methyl sterols at 20°C, but not Δ7-dafachronic acid (N≥6, M ± SEM; **below detection limit, *p<0.05). (B) Levels of lathosterone, 4-cholesten-3-one, and Δ7-dafachronic acid at the elevated temperature of 25°C relative to N2 wild-type. No significant change in lathosterone or 4-cholesten-3-one is noted in hsd-1 single mutants, whereas DA levels are elevated relative to wild-type. dhs-16;hsd-1 double mutants display decreased levels of DA, corresponding with the more severe L2d and dauer phenotypes displayed by these animals, but show increased levels of 4-cholesten-3-one, presumably due to feedback (N≥3, M ± SEM; **below detection limit, *p<0.05). (TIF) [file pbio.1001305.s004.tif]

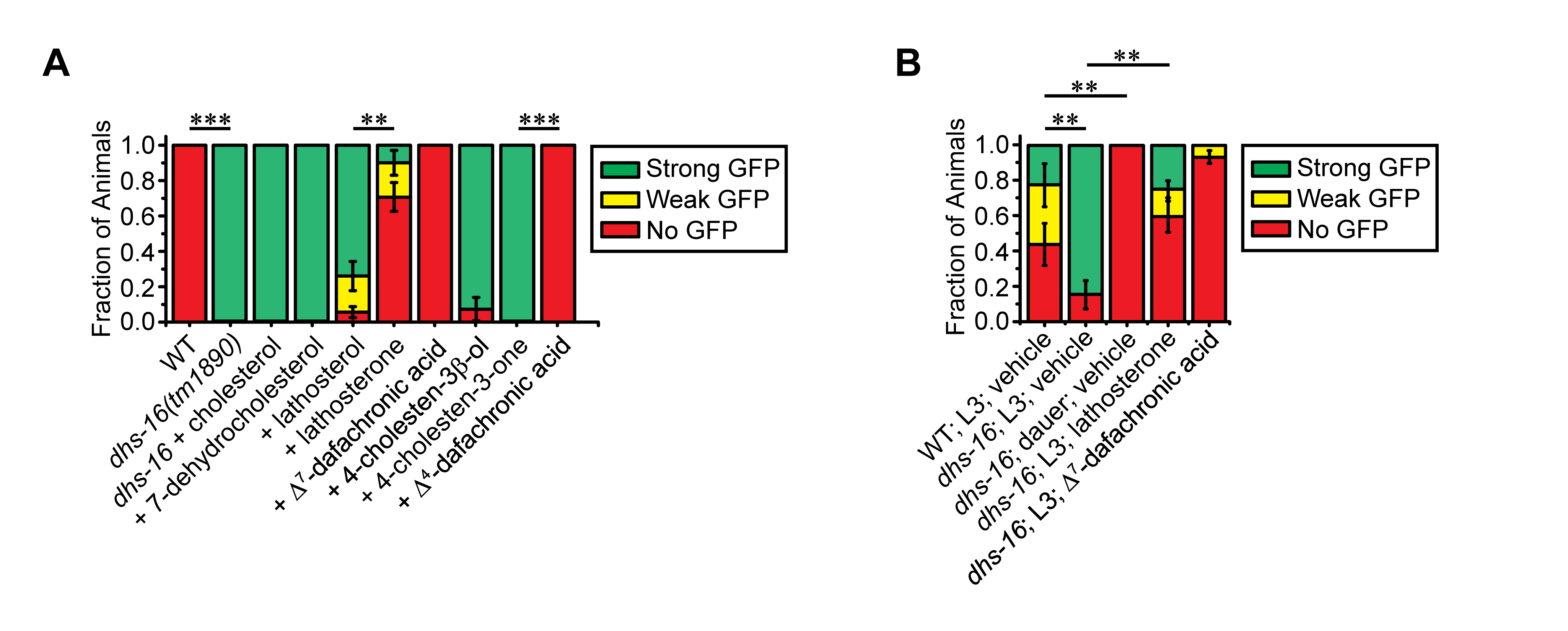

Supplement: Figure S5 — Homeostatic feedback on hypodermal daf-9 expression. (A) daf-9::gfp hypodermal expression is upregulated in dhs-16(tm1890) mutants at 20°C (***p<0.0001). Lathosterone and the DAs fully rescue this upregulation, while proposed upstream precursors do not (**p<0.01). The fraction of animals with strong (green), weak (yellow), or no (red) hypodermal GFP expression is shown (N≥3, M ± SD). (B) At 27°C, dhs-16 mutant animals enter dauer diapause, in which daf-9::gfp hypodermal expression is downregulated. Animals that do not enter dauer display higher levels of daf-9 expression than N2 wild-type, as seen at 20°C (**p<0.01). Feeding lathosterone rescues the dauer phenotype and restores daf-9::gfp expression to wild-type levels (N≥3, M ± SD; **p<0.01). (TIF) [file pbio.1001305.s005.tif]

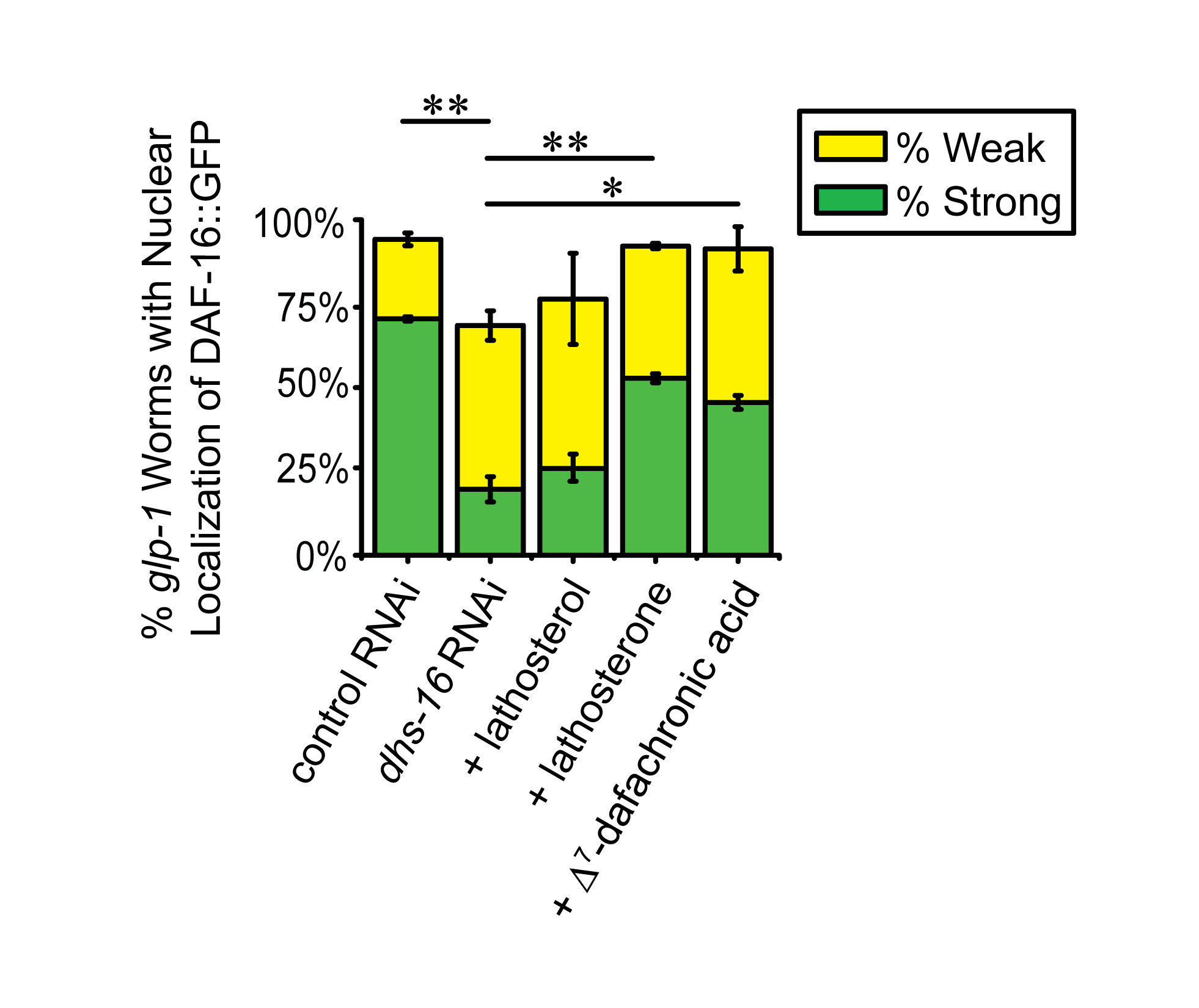

Supplement: Figure S6 — RNAi knockdown of dhs-16 reduces DAF-16::GFP localization in germline-less mutant animals. Treatment of glp-1(e2141ts) animals with dhs-16 RNAi leads to a reduction in strongly nuclear-localized intestinal DAF-16::GFP in day 1 adults at the restrictive temperature of 25°C. Percent animals with strong localization (green) and weak localization (yellow) are displayed. Localization is restored upon provision of lathosterone or Δ7-dafachronic acid, but not with lathosterol (N = 3, M ± SD; **p<0.01, *p<0.05). (TIF) [file pbio.1001305.s006.tif]
