# Supplementary material for: A Novel 3-Hydroxysteroid Dehydrogenase That Regulates Reproductive Development and Longevity
Source: PLoS Biol. 2012 Apr 10;10(4):e1001305. doi: 10.1371/journal.pbio.1001305 (PMC3323522; doi:10.1371/journal.pbio.1001305)
Supplement: Table S1 — Knockdown of emb-8 leads to DA deficiency-associated phenotypes. (DOC) [file pbio.1001305.s007.doc]

Table S1. Knockdown of *emb-8* leads to DA Deficiency-Associated Phenotypes

| **Genotype** | **Daf-c at 27°C±SE(%)^a^** | **N^b^** | **Mig at 25°C±SE(%)^c^** | **N^b^** |
| --- | --- | --- | --- | --- |
| WT; control RNAi | 38±4 | 1261(6) | 0±0 | 420(11) |
| WT; *daf-36* RNAi | 67±6 | 1397(4) | nd |  |
| WT; *daf-9* RNAi | 61±6 | 1309(6) | 1±1 | 76(2) |
| WT; *dhs-16* RNAi | 70±3 | 1481(6) | 2±1 | 136(3) |
| WT; *emb-8* RNAi | 77±8 | 1761(5) | 2±1 | 276(7) |
| *daf-36(k114)*; control RNAi | nd |  | 2±1 | 114(3) |
| *daf-36(k114); daf-9* RNAi | nd |  | 52±6 | 188(3) |
| *daf-36(k114); dhs-16* RNAi | nd |  | 49±5 | 268(3) |
| *daf-36(k114); emb-8* RNAi | nd |  | 66±13 | 134(3) |
| *dhs-16(tm1890)*; control RNAi | nd |  | 0±0 | 90(3) |
| *dhs-16(tm1890); emb-8* RNAi | nd |  | 55±1 | 120(3) |
| *daf-9(k182)*; control RNAi | nd |  | 9±6 | 72(2) |
| *daf-9(k182); emb-8* RNAi | nd |  | 70±12 | 74(2) |
| *daf-16(mu86)* | 23±5 | 974(3) | nd |  |
| *daf-16(mu86); emb-8* RNAi | 60±6 | 1159(3) | nd |  |
| *daf-5(e1386)* | 48±18 | 1259(3) | nd |  |
| *daf-5(e1386); emb-8* RNAi | 62±15 | 1319(3) | nd |  |
| *daf-12(rh61rh411);* control RNAi | 0±0 | 946(3) | 0±0 | 80(2) |
| *daf-12(rh61rh411); emb-8* RNAi | 0±0 | 1111(3) | 1±1 | 80(2) |
| *daf-36; daf-12;* control RNAi | nd |  | 1±1 | 138(3) |
| *daf-36; daf-12; emb-8* RNAi | nd |  | 0±0 | 114(3) |
| *dhs-16;daf-12;* control RNAi | nd |  | 1±1 | 120(3) |
| *dhs-16; daf-12;* *emb-8* RNAi | nd |  | 2±1 | 122(3) |
| *daf-9(dh6); daf-12*; control RNAi | nd |  | 2±2 | 122(3) |
| *daf-9(dh6);daf-12; emb-8* RNAi | nd |  | 4±2 | 119(3) |

^a^ Dauers formed under reproductive growth conditions.

^b^ Number of experiments given in parentheses.

^c^ Hermaphrodite distal tip cells that fail to turn in L3, n > 50 cells.
