# Supplementary material for: A Novel 3-Hydroxysteroid Dehydrogenase That Regulates Reproductive Development and Longevity
Source: PLoS Biol. 2012 Apr 10;10(4):e1001305. doi: 10.1371/journal.pbio.1001305 (PMC3323522; doi:10.1371/journal.pbio.1001305)
Supplement: Table S2 — Longevity in the absence of the germline is partially dependent upon dhs-16. (DOC) [file pbio.1001305.s008.doc]

Table S2. Longevity in the Absence of the Germline is Partially Dependent upon *dhs-16*

| **Exp#** | **Genotype** | **Treatment** | **Mean Lifespan(Days)** | **Max Lifespan**  **(Days)** | **N (Observed/Total)** | ***P* (versus Mock Ablated)** | ***P* (versus WT Ablated)** |
| --- | --- | --- | --- | --- | --- | --- | --- |
| **1** | N2 | Mock | 23.6 | 31 | 41/60 |  |  |
| **1** | N2 | Ablated | 45.9 | 66 | 41/57 | <0.0001 |  |
| **1** | *dhs-16(tm1890)* | Mock | 21.5 | 31 | 33/60 |  |  |
| **1** | *dhs-16(tm1890)* | Ablated | 21.3 | 48 | 49/66 | 0.6935 | <0.0001 |
| **2** | N2 | Mock | 22.2 | 34 | 51/105 |  |  |
| **2** | N2 | Ablated | 39.7 | 63 | 42/62 | <0.0001 |  |
| **2** | *dhs-16(tm1890)* | Mock | 18.5 | 30 | 26/102 |  |  |
| **2** | *dhs-16(tm1890)* | Ablated | 16.7 | 51 | 47/66 | 0.0154 | <0.0001 |
