# Supplementary material for: A Novel 3-Hydroxysteroid Dehydrogenase That Regulates Reproductive Development and Longevity
Source: PLoS Biol. 2012 Apr 10;10(4):e1001305. doi: 10.1371/journal.pbio.1001305 (PMC3323522; doi:10.1371/journal.pbio.1001305)
Supplement: Table S3 — Settings for LC/MS/MS analysis of DA precursors. (DOC) [file pbio.1001305.s009.doc]

Table S3. Settings for LC/MS/MS Analysis of DA Precursors

| **Compound** | **Fragmentor Voltage** | **Collision Energy** |
| --- | --- | --- |
| cholesterol-*d_7_* | 105 | 1 |
| lathosterol | 100 | 3 |
| 7-dehydrocholesterol | 100 | 3 |
| lathosterone | 125 | 7 |
| 4-cholesten-3-one | 215 | 30 |
